# Supplementary material for: Unraveling the rate-determining step of C2+ products during electrochemical CO reduction
Source: Nat Commun. 2024 Jan 30;15:892. doi: 10.1038/s41467-024-45230-1 (PMC10828390; doi:10.1038/s41467-024-45230-1)
Supplement: Supplementary file 1 — Supplementary Information [file 41467_2024_45230_MOESM1_ESM.pdf]

Supplementary Information

Unraveling the rate-determining step of C_{2+} products during electrochemical CO reduction

Wanyu Deng[a,b,c], Peng Zhang[a,b], Yu Qiao[c], Georg Kastlunger[c], Nitish Govindarajan[c], Aoni Xu[c], Ib Chorkendorff[c], Brian Seger[c,*], and Jinlong Gong[a,b,*]

[a]*Key Laboratory for Green Chemical Technology of Ministry of Education, School of Chemical Engineering and Technology, Tianjin University, Collaborative Innovation Center of Chemical Science and Engineering (Tianjin), Tianjin 300072, China*

[b]*Joint School of National University of Singapore and Tianjin University, International Campus of Tianjin University, Binhai New City, Fuzhou 350207, China*

[c]*Department of Physics, Technical University of Denmark, 2800 Kgs. Lyngby, Denmark*

*Corresponding Author: jlgong@tju.edu.cn; brse@fysik.dtu.dk

Supplementary Note 1: Derivation of mechanistic models

By analyzing the RDSs, the preceding and following reaction processes can be speculated, which are detailed in Table 1 and Table 2. Specifically, for the *CO(CO) coupling reaction mechanism (Table 1), one only needs to consider the initial CO adsorption process, as any C_{2+} intermediate produced after C-C coupling will create C_{2+} products. However, when assuming *CO protonation as RDSs, it is still necessary to consider whether its subsequent C_{2+} product generation is via CO-*CO(H) coupling, *CO-*CO(H) coupling, or *CO(H)-*CO(H) coupling. Thus, there are three possible reaction mechanisms for the same RDS step in Table 2. In addition, the key intermediate of *CO(H), generated by *CO hydrogenation, is also an intermediate for CH_4. Therefore, the subsequent CH_4 reaction process should also be included in Table 2.

Mechanism 1: the rate-determining step (RDS) is two (*)CO coupling (Table 1):

One, the coupling between two *CO.

A1 $\quad CO + * \rightarrow *CO$

A2 $\quad *CO + *CO + e^{\delta-} \xrightarrow{RDS} *C_2O_2^{\delta-} + *$

The theoretical current density of this process can be written as:

$$j_{C2+} = k_{A2}^0[*\mathrm{CO}]^2 \exp(-\alpha f \eta) \tag{S1}$$

In Eqn. S1, j is the current density and C_{2+} in j_{C2+} represents the j of C_{2+} products; k^0 is the standard forward rate constant and A2 in k_{A2}^0 represents the k^0 of step A2; [*CO] is the concentration of adsorbed CO; α is the transfer coefficient assumed to be equal to 0.5; $f = F/RT$, where R is the ideal gas constant, T is the absolute temperature, F is the Faraday constant; and η is the overpotential for the cathodic reaction.

Here, the CO adsorption is assumed to be a fast equilibrium step. The following equation can be given:

$$K_{*CO}^0 = \frac{[*\mathrm{CO}]}{H_{CO}P_{CO}[*]} \tag{S2}$$

In Eqn. S2, K_{*CO}^0 is the standard equilibrium constant of step CO adsorption; H_{CO} is

the Henry's law constants of CO gas; P_{CO} is the pressure of CO gas; $*$ is the vacant active site.

Because *CO is the only major adsorbate, the concentration of all sites [L] can be written as:

$$[*]+[*\mathrm{CO}]=[\mathrm{L}] \tag{S3}$$

From Eqn. S2 and S3, we can obtain:

$$[*\mathrm{CO}]=\frac{K_{*CO}^{0}H_{CO}P_{CO}[\mathrm{L}]}{1+K_{*CO}^{0}P_{CO}} \tag{S4}$$

Combining Eqn. S4 and S1 yields:

$$j_{C2+}=k_{A2}^{0}\left(\frac{K_{*CO}^{0}H_{CO}P_{CO}[\mathrm{L}]}{1+K_{*CO}^{0}H_{CO}P_{CO}}\right)^{2}\exp\left(-\alpha f\eta\right) \tag{S5}$$

Which can be simplified as

$$j_{C2+}=\frac{a_A P_{CO}{}^{2}}{\left(b_A P_{CO}+1\right)^{2}} \tag{S6}$$

$$a_A=k_{A2}^{0}\exp\left(-\alpha f\eta\right)K_{*CO}^{0}{}^{2}[\mathrm{L}]^{2}H_{CO}{}^{2}$$
$$b_A=H_{CO}K_{*CO}^{0}$$

The reaction order of CO (n_{CO}) can be calculated by

$$n_{CO}\equiv P_{CO}\frac{\partial\ln j_{C2+}}{\partial P_{CO}} \tag{S7}$$

Combining Eqn. S5 and S7 yields:

$$n_{CO}=\frac{2}{b_A P_{CO}+1} \tag{S8}$$

Therefore, **the n_{CO} is between 2 to 0 (2 ~ 0) from low pressure ($P_{CO}\ll\frac{1}{b_A}$) to high pressure ($P_{CO}\gg\frac{1}{b_A}$)**. Due to experimental limitations, we were unable to conduct experiments under higher pressure conditions than 1 atm. Therefore, we employed a nonlinear fitting approach to determine the reaction order of CO, which effectively mitigated the influence of pressure limitations on our result analysis. However, to simplify the rate expression to observe the reaction order of other reactants such as

H^+. This simplification does not affect our final conclusions.

From Eqn. S5, **the reaction orders of H^+ (n_{H^+}) and H_2O (n_{H_2O}) are both 0.** A deeper understanding of the n_{CO} suggests that as the coverage of CO on the catalyst surface increases to saturation, further increasing the CO partial pressure does not accelerate the reaction rate. This phenomenon is reflected in the reaction order of 0 for CO.

Two, the coupling between *CO and CO.

B1 $\quad CO + * \rightarrow *CO$

B2 $\quad *CO + CO + e^{\delta-} \xrightarrow{RDS} *C_2O_2^{\delta-}$

Similar to the process of the coupling between two *CO, this proposed reaction process can list the following equations:

$$j_{C2+} = k_{B2}^0 H_{CO}[*\mathrm{CO}]P_{CO}\exp(-\alpha f\eta) \tag{S9}$$

$$K_{*CO}^0 = \frac{[*\mathrm{CO}]}{H_{CO}P_{CO}[*]} \tag{S10}$$

$$[*]+[*\mathrm{CO}]=[\mathrm{L}] \tag{S11}$$

Combining Eqn. S9 to S11 yields:

$$j_{C2+} = k_{B2}^0 \frac{K_{*CO}^0 H_{CO}{}^2 P_{CO}{}^2[\mathrm{L}]}{1+K_{*CO}^0 H_{CO}P_{CO}}\exp(-\alpha f\eta) \tag{S12}$$

Which can be simplified as

$$j_{C2+} = \frac{a_B P_{CO}{}^2}{1+b_B P_{CO}} \tag{S13}$$

$$a_B = k_{B2}^0 K_{*CO}^0 H_{CO}{}^2[\mathrm{L}]\exp(-\alpha f\eta)$$
$$b_B = K_{*CO}^0 H_{CO}$$

Combining Eqn. S7 and S13 yields:

$$n_{co} = 2 - \frac{b_B P_{CO}}{b_B P_{CO}+1} \tag{S14}$$

Therefore, **the n_{CO} is 2 ~ 1 from low pressure ($P_{CO} \ll \frac{1}{b_B}$)to high pressure ($P_{CO} \gg \frac{1}{b_B}$).**

From Eqn. S12, **the n_{H^+} and n_{H_2O} are both 0.**

Mechanism 2: the RDS is the protonation to *CO (Table 2):

One: the proton source is the H_2O molecule.

C1		$CO + * \rightarrow *CO$
C2		$*CO + H_2O + e^- \xrightarrow{RDS} *CO(H) + OH^-$
	C3.1	$CO + *CO(H) \rightarrow *C_2O_2(H)$
C3	C3.2	$*CO + *CO(H) \rightarrow *C_2O_2(H) + *$
	C3.3	$*CO(H) + *CO(H) \rightarrow *C_2O_2(H)_2 + *$
C4		$*CO(H) + H_2O + e^- \rightarrow *CO(H)_2 + OH^-$

The theoretical current density of this process can be written as:

$$j_{C2+} + j_{CH4} = k_{C2}^{0}[*\mathrm{CO}][\mathrm{H_2O}]\exp(-\alpha f\eta) \quad \text{(S15)}$$

The current density of C2+ can be written as:

$$j_{C2+} = (j_{C2+} + j_{CH4}) * S_{C2+} \quad \text{(S16)}$$

S_{C2+} is the selectivity of C_{2+} products over carbon-related products during COER.

Here, the CO adsorption is assumed to be a fast equilibrium step. The following equation can be given:

$$K_{*CO}^{0} = \frac{[*\mathrm{CO}]}{H_{CO}P_{CO}[*]} \quad \text{(S17)}$$

Being produced in the RDS of the reaction network, [*CO(H)] should be small due to its consumption being faster than its production. Therefore, *CO is the only major adsorbate and the concentration of all sites [L] can be expressed as:

$$[*] + [*\mathrm{CO}] = [L] \quad \text{(S18)}$$

From Eqn. S17 and S18, the following equation can be obtained:

$$[{}^*\mathrm{CO}]=\frac{K^0_{*CO}H_{CO}P_{CO}[L]}{1+K^0_{*CO}H_{CO}P_{CO}} \quad \text{(S19)}$$

Therefore, the final rate expression of C_{2+} formation can be written as:

$$j_{C2+}=S_{C2+}k^0_{C2}\frac{K^0_{*CO}H_{CO}P_{CO}}{1+K^0_{*CO}H_{CO}P_{CO}}[L][\mathrm{H_2O}]\exp(-\alpha f\eta) \quad \text{(S20)}$$

Which can be simplified as

$$j_{C2+}=\frac{a_C P_{CO}}{b_C P_{CO}+1} \quad \textbf{(S21)}$$

$$a_{C3}=S_{C2+}k^0_{C2}K^0_{*CO}H_{CO}[L][\mathrm{H_2O}]\exp(-\alpha f\eta)$$

$$b_{C3}=K^0_{*CO}H_{CO}$$

Combining Eqn. S7 and S21 yields:

$$n_{CO}=\frac{1}{1+b_C P_{CO}} \quad \text{(S22)}$$

Therefore, **the** n_{CO} **is 1 ~ 0 from low pressure (**$P_{CO}\ll\frac{1}{b_C}$**) to high pressure (**$P_{CO}\gg\frac{1}{b_C}$**).**

At high P_{CO}, according to Eqn. S20, the rate expression can be expressed as:

$$j_{C2+}=S_{C2+}k^0_{C2}[L][\mathrm{H_2O}]\exp(-\alpha f\eta) \quad \text{(S23)}$$

Thus, the n_{H^+} **and** n_{H_2O} **are 0 and 1, respectively.**

At low P_{CO}, according to Eqn. S20, the rate expression can be expressed as:

$$j_{C2+}=S_{C2+}k^0_{C2}K^0_{*CO}H_{CO}P_{CO}[L][\mathrm{H_2O}]\exp(-\alpha f\eta) \quad \text{(S24)}$$

Thus, the n_{H^+} **and** n_{H_2O} **are 0 and 1, respectively.**

Two: the proton source is the H^+.

D1		$CO + {}^* \rightarrow {}^*CO$
D2		${}^*CO + H^+ + e^- \xrightarrow{\text{RDS}} {}^*CO(H)$
D3	D3.1	$CO + {}^*CO(H) \rightarrow {}^*C_2O_2(H)$

D3.2 *CO + *CO(H) → *C_2O_2(H) + *

D3.3 *CO(H) + *CO(H) → *$C_2O_2(H)_2$ + *

D4 *CO(H) + H^+ + e^- → *$CO(H)_2$

The theoretical current density of this process can be written as:

$$j_{C2+} + j_{CH4} = k_{D2}^{0}[\text{*CO}][\text{H}^{+}]\exp(-\alpha f\eta) \quad \text{(S25)}$$

Combining Eqn. S16, S19, and S25, the final rate expression of C_{2+} formation can be written as:

$$j_{C2+} = S_{C2+}k_{C2}^{0}\frac{K_{*CO}^{0}H_{CO}P_{CO}}{1+K_{*CO}^{0}H_{CO}P_{CO}}[L][\text{H}^{+}]\exp(-\alpha f\eta) \quad \text{(S26)}$$

Which can be simplified as

$$j_{C2+} = \frac{a_D P_{CO}}{b_D P_{CO}+1} \quad \textbf{(S27)}$$

$$a_D = S_{C2+}k_{D2}^{0}K_{*CO}^{0}H_{CO}[L][\text{H}^{+}]\exp(-\alpha f\eta)$$

$$b_D = K_{*CO}^{0}H_{CO}$$

Combining Eqn. S7 and S27 yields:

$$n_{CO} = \frac{1}{1+b_D P_{CO}} \quad \text{(S28)}$$

Therefore, **the n_{CO} is 1 ~ 0 from low pressure ($P_{CO} \ll \frac{1}{b_D}$) to high pressure ($P_{CO} \gg \frac{1}{b_D}$).**

At high P_{CO}, according to Eqn. S26, the rate expression can be expressed as:

$$j_{C2+} = S_{C2+}k_{D2}^{0}[L][\text{H}^{+}]\exp(-\alpha f\eta) \quad \text{(S29)}$$

Thus, the n_{H^+} and n_{H_2O} are 1 and 0, respectively.

At low P_{CO}, according to Eqn. S26, the rate expression can be expressed as:

$$j_{C2+} = S_{C2+}k_{D2}^{0}K_{*CO}^{0}H_{CO}P_{CO}[L][\text{H}^{+}]\exp(-\alpha f\eta) \quad \text{(S30)}$$

Thus, the n_{H^+} and n_{H_2O} are 1 and 0, respectively.

Three: the proton source is the *H from H_2O.

E1		$CO + * \rightarrow *CO$
E2		$H_2O + * + e^- \rightarrow *H + OH^-$
E3		$*CO + *H \xrightarrow{RDS} *CO(H) + *$
	E4.1	$CO + *CO(H) \rightarrow *C_2O_2(H)$
E4	E4.2	$*CO + *CO(H) \rightarrow *C_2O_2(H) + *$
	E4.3	$*CO(H) + *CO(H) \rightarrow *C_2O_2(H)_2 + *$
E5		$*CO(H) + *H \rightarrow *CO(H)_2 + *$

The theoretical current density of this process can be written as:

$$j_{C2+} + j_{CH4} = k_{E3}^0[*\mathrm{CO}][*\mathrm{H}] \quad \text{(S31)}$$

Here, the CO adsorption and H_2O dissociation are assumed to be fast equilibrium steps. The following equation can be given:

$$K_{*CO}^0 = \frac{[*\mathrm{CO}]}{H_{CO}P_{CO}[*]} \quad \text{(S32)}$$

$$K_{E2}^0 = \frac{[*\mathrm{H}][\mathrm{OH}^-]}{[\mathrm{H_2O}][*]\exp(-f\eta)} \quad \text{(S33)}$$

[*CO(H)] should be small due to its consumption being faster than its production. Therefore, *CO and *H are the major adsorbates and the concentration of all sites [L] can be expressed as:

$$[*]+[*\mathrm{CO}]+[*\mathrm{H}]=[L] \quad \text{(S34)}$$

From Eqn. S32 to S34, the following equation can be obtained:

$$[*\mathrm{CO}] = \frac{K_{*CO}^0 H_{CO}P_{CO}[\mathrm{L}][\mathrm{OH}^-]}{[\mathrm{OH}^-]+K_{*CO}^0 H_{CO}P_{CO}[\mathrm{OH}^-]+K_{E2}^0[\mathrm{H_2O}]\exp(-f\eta)} \quad \text{(S35)}$$

$$[*\mathrm{H}] = \frac{K_{E2}^0[\mathrm{H_2O}][\mathrm{L}]\exp(-f\eta)}{[\mathrm{OH}^-]+K_{*CO}^0 H_{CO}P_{CO}[\mathrm{OH}^-]+K_{E2}^0[\mathrm{H_2O}]\exp(-f\eta)} \quad \text{(S36)}$$

Combining Eqn. S16, S31, S35 and S36, the rate expression of C_{2+} formation is:

$$j_{C2+} = \frac{S_{C2+}k_{E3}^0 K_{E2}^0 K_{*CO}^0 H_{CO}P_{CO}[\mathrm{L}]^2[\mathrm{H_2O}][\mathrm{OH}^-]\exp(-f\eta)}{([\mathrm{OH}^-]+K_{*CO}^0 H_{CO}P_{CO}[\mathrm{OH}^-]+K_{E2}^0[\mathrm{H_2O}]\exp(-f\eta))^2}$$

(S37)

Which can be simplified as

$$j_{C2+} = \frac{a_E P_{CO}}{\left(b_E P_{CO} + 1\right)^2} \quad \textbf{(S38)}$$

$$a_E = \frac{S_{C2+} k_{E3}^0 K_{E2}^0 K_{*CO}^0 H_{CO} [\mathrm{L}]^2 [\mathrm{H_2O}][\mathrm{OH^-}]\exp(-f\eta)}{([\mathrm{OH^-}] + K_{E2}^0 [\mathrm{H_2O}]\exp(-f\eta))^2}$$

$$b_E = \frac{K_{*CO}^0 H_{CO} [\mathrm{OH^-}]}{[\mathrm{OH^-}] + K_{E2}^0 [\mathrm{H_2O}]\exp(-f\eta)}$$

Combining Eqn. S7 and S38 yields:

$$n_{CO} = \frac{1 - b_E P_{CO}}{b_E P_{CO} + 1} \quad \text{(S39)}$$

Therefore, **the** n_{CO} **is 1 ~ −1 from low pressure (**$P_{CO} \ll \frac{1}{b_{E4}}$**)to high pressure (**$P_{CO} \gg \frac{1}{b_{E4}}$**).**

At high P_{CO}, according to Eqn. S37, the rate expression can be expressed as:

$$j_{C2+} = \frac{S_{C2+} k_{E3}^0 K_{E2}^0 [\mathrm{L}]^2 [\mathrm{H^+}]\exp(-f\eta)}{K_W^0 K_{*CO}^0 H_{CO} P_{CO}} \quad \text{(S40)}$$

In Eqn. S40, K_W^0 is the standard equilibrium constant of H_2O dissociation. Here, **the** n_{H^+} **and** n_{H_2O} **are 1 and 0, respectively.**

At low P_{CO}, according to Eqn. S37, the rate expression can be expressed as:

$$j_{C2+} = \frac{S_{C2+} k_{E3}^0 K_{E2}^0 K_{*CO}^0 H_{CO} [\mathrm{L}]^2 [\mathrm{H^+}] K_W^0 \exp(-f\eta) P_{CO}}{(K_W^0 + [\mathrm{H^+}] K_{E2}^0 \exp(-f\eta))^2} \quad \text{(S41)}$$

When $K_W^\theta \gg K_{E2}^\theta \exp(-f\eta)[\mathrm{H^+}]$,

$$j_{C2+} = \frac{S_{C2+} k_{E3}^0 K_{E2}^0 K_{*CO}^0 H_{CO} [\mathrm{L}]^2 [\mathrm{H^+}]\exp(-f\eta) P_{CO}}{K_W^0} \quad \text{(S42)}$$

Thus, **the** n_{H^+} **and** n_{H_2O} **are 1 and 0, respectively.**

When $K_W^0 \ll K_{E2}^0 [\mathrm{H_2O}]\exp(-f\eta)[\mathrm{H^+}]$,

$$j_{C2+} = \frac{S_{C2+}k_{E3}^0 K_{*CO}^0 H_{CO}[\mathrm{L}]^2 K_W^0 P_{CO}}{K_{E2}^0[\mathrm{H}^+]\exp(-f\eta)} \tag{S43}$$

Thus, **the** n_{H^+} **and** n_{H_2O} **are -1 and 0, respectively.**

Four: the proton source is the *H from H^+.

F1		$CO + * \rightarrow *CO$
F2		$H^+ + * + e^- \rightarrow *H$
F3		$*CO + *H \xrightarrow{\mathrm{RDS}} *CO(H) + *$
F4	F4.1	$CO + *CO(H) \rightarrow *C_2O_2(H)$
	F4.2	$*CO + *CO(H) \rightarrow *C_2O_2(H) + *$
	F4.3	$*CO(H) + *CO(H) \rightarrow *C_2O_2(H)_2 + *$
F5		$*CO(H) + *H \rightarrow *CO(H)_2 + *$

The theoretical current density of this process can be written as:

$$j_{C2+} + j_{CH4} = k_{E3}^0[*\mathrm{CO}][*\mathrm{H}] \tag{S44}$$

Here, the CO and H^+ adsorption are assumed to be fast equilibrium steps. The following equation can be given:

$$K_{*CO}^0 = \frac{[*\mathrm{CO}]}{H_{CO}P_{CO}[*]} \tag{S45}$$

$$K_{E2}^0 = \frac{[*\mathrm{H}]}{[\mathrm{H}^+][*]\exp(-f\eta)} \tag{S46}$$

[*CO(H)] should be small due to its consumption being faster than its production. Therefore, *CO and *H are the major adsorbates and the concentration of all sites [L] can be expressed as:

$$[*]+[*\mathrm{CO}]+[*\mathrm{H}]=[L] \tag{S47}$$

From Eqn. S45 to S47S, the following equation can be obtained:

$$[*\mathrm{CO}] = \frac{K_{*CO}^0 H_{CO}P_{CO}[\mathrm{L}]}{1+K_{*CO}^0 H_{CO}P_{CO} + K_{F2}^0[\mathrm{H}^+]\exp(-f\eta)} \tag{S48}$$

$$[*\mathrm{H}] = \frac{K_{F2}^0[\mathrm{H}^+][\mathrm{L}]\exp(-f\eta)}{1+K_{*CO}^0 H_{CO}P_{CO} + K_{F2}^0[\mathrm{H}^+]\exp(-f\eta)} \tag{S49}$$

Combining Eqn. S16, the rate expression of C_{2+} formation can be written as:

$$j_{C2+}=\frac{S_{C2+}k_{E3}^{0}K_{F2}^{0}K_{*CO}^{0}H_{CO}P_{CO}[\mathrm{L}]^{2}[\mathrm{H}^{+}]\exp(-f\eta)}{(1+K_{*CO}^{0}H_{CO}P_{CO}+K_{F2}^{0}[\mathrm{H}^{+}]\exp(-f\eta))^{2}} \tag{S50}$$

Which can be simplified as

$$j_{C2+}=\frac{a_F P_{CO}}{\left(b_F P_{CO}+1\right)^{2}} \tag{S51}$$

$$a_F=\frac{S_{C2+}k_{E3}^{0}K_{F2}^{0}K_{*CO}^{0}H_{CO}[\mathrm{L}]^{2}[\mathrm{H}^{+}]\exp(-f\eta)}{(1+K_{F2}^{0}[\mathrm{H}^{+}]\exp(-f\eta))^{2}}$$

$$b_F=\frac{K_{*CO}^{0}H_{CO}}{1+K_{F2}^{0}[\mathrm{H}^{+}]\exp(-f\eta)}$$

Combining Eqn. S7 and S51 yields:

$$n_{CO}=\frac{1-b_F P_{CO}}{b_F P_{CO}+1} \tag{S52}$$

Therefore, **the** n_{CO} **is 1 ~ −1 from low pressure (** $P_{CO}\ll\frac{1}{b_F}$ **) to high pressure (** $P_{CO}\gg\frac{1}{b_F}$ **).**

At high P_{CO}, according to Eqn. S50, the rate expression can be expressed as:

$$j_{C2+}=\frac{S_{C2+}k_{E3}^{0}K_{F2}^{0}[\mathrm{L}]^{2}[\mathrm{H}^{+}]\exp(-f\eta)}{K_{*CO}^{0}H_{CO}P_{CO}} \tag{S53}$$

Thus, **the** n_{H^+} **and** n_{H_2O} **are 1 and 0, respectively.**

At low P_{CO}, according to Eqn. S50, the rate expression can be expressed as:

$$j_{C2+}=\frac{S_{C2+}k_{E3}^{0}K_{F2}^{0}K_{*CO}^{0}H_{CO}P_{CO}[\mathrm{L}]^{2}[\mathrm{H}^{+}]\exp(-f\eta)}{(1+K_{F2}^{0}[\mathrm{H}^{+}]\exp(-f\eta))^{2}} \tag{S54}$$

When $1\gg K_{F2}^{0}[\mathrm{H}^{+}]\exp(-f\eta)$,

$$j_{C2+}=S_{C2+}k_{E3}^{0}K_{F2}^{0}K_{*CO}^{0}H_{CO}P_{CO}[\mathrm{L}]^{2}[\mathrm{H}^{+}]\exp(-f\eta) \tag{S55}$$

Thus, **the** n_{H^+} **and** n_{H_2O} **are 1 and 0, respectively.**

When $1 \ll K_{F2}^{0}[\mathrm{H}^{+}]\exp(-f\eta)$,

$$j_{C2+} = \frac{S_{C2+} k_{E3}^{0} K_{*CO}^{0} H_{CO} P_{CO} [\mathrm{L}]^{2}}{K_{F2}^{0} \exp(-f\eta) [\mathrm{H}^{+}]} \tag{S56}$$

Thus, the n_{H^+} and n_{H_2O} are −1 and 0, respectively.

Supplementary Note 2: Simulated hydrogen coverage on Cu under reaction conditions

We calculated the reaction kinetics for hydrogen evolution reaction (HER) via constant potential DFT simulations in order to estimate the hydrogen coverage on the catalyst surface ([H]) needed for the reaction order derivations. All calculations have been performed using the Solvated Jellium method (SJM)[1], implemented in GPAW[2,3]. The calculation setup and methods are identical to the ones described in Reference[4]. The base data and simulation routines are provided free of charge at https://gitlab.com/gkastlun/h_coverage_on_cu.

Both the kinetic barriers of the Volmer-Heyrovsky (V-H) and Volmer-Volmer-Tafel (V-V-T) mechanisms in alkaline conditions (with H_2O as proton donor) were estimated and used in the microkinetic simulations within the CatMap code.[5] The Cu(100) and Cu(211) facets were studied in the present study, as the former is often considered the most active facet for *CO-*CO dimerization[4,6], while the latter is considered the most active facet for CH_4 production[4,7]. Supplementary Fig. 1 shows the determined free energy diagrams at a potential of −1.2 V vs SHE and a pH of 13. Note that we approximated 0 V vs SHE as a work function of 4.4 eV in our simulations[4,8].

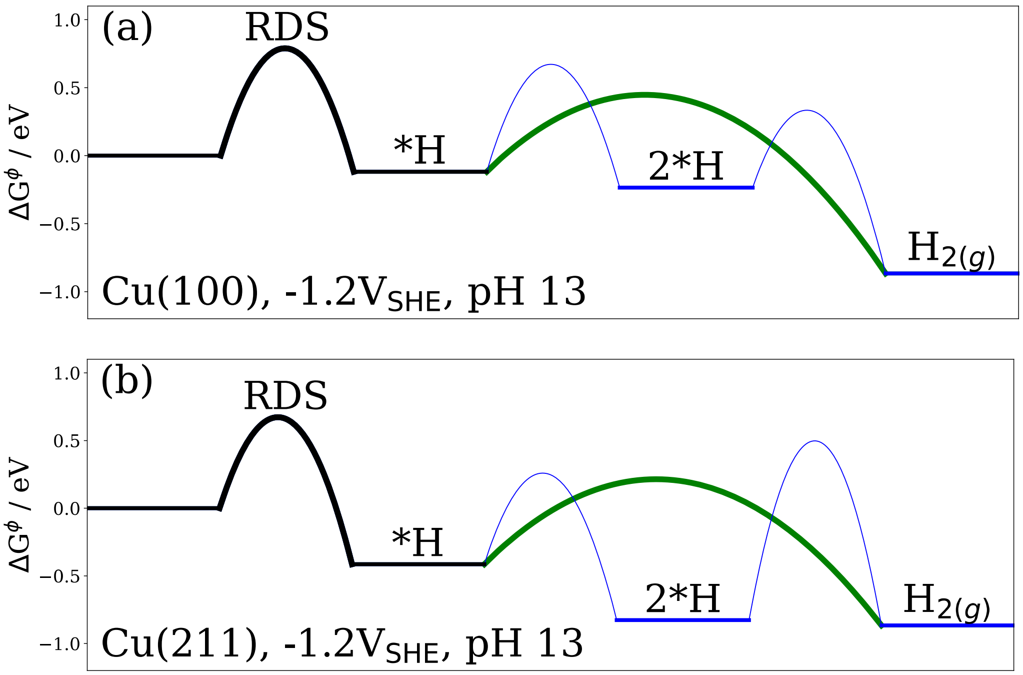

Supplementary Fig. 1. Calculated free energy diagrams of HER on (a) Cu(100) and (b) Cu(211) at −1.2V vs SHE and pH 13. The Heyrovsky step is shown in green, while the steps relevant for the V-T mechanism are shown in blue. The preferred mechanism is highlighted in bold. Note that on both shown facets the Volmer step represents the rate limiting step as it has the highest barriers.

We identified the mechanism of HER on both Cu(100) and Cu(211) in alkaline conditions to proceed via the V-H mechanism, with Volmer as the RDS. However, we note that the Volmer and Heyrovsky barriers are comparable on Cu(211). Thus, an intermediate coverage of *H is expected on the 211 surface.

By including the calculated reaction energetics in a microkinetic model, we simulated the steady state H-coverages, which we show in Supplementary Fig. 2. We find that on the (100) facet, shown in Supplementary Fig. 2a, the coverage stays well below 1% over the potential and pH ranges of interest with a slight increase at higher potentials. On the (211) step, shown in Supplementary Fig. 2b, on the other hand, a coverage of about 10% is present at reaction conditions, which reduces with increasing cathodic potentials. The reduction in coverage with potential is a consequence of the larger potential response estimated for the Heyrovsky step (α=0.43) compared the Volmer step (α=0.36) from the

constant-potential DFT simulations. The coverage reaches a maximum at low pH and potentials slightly more negative than the equilibrium potential of *H adsorption (−0.05 V_{RHE}).

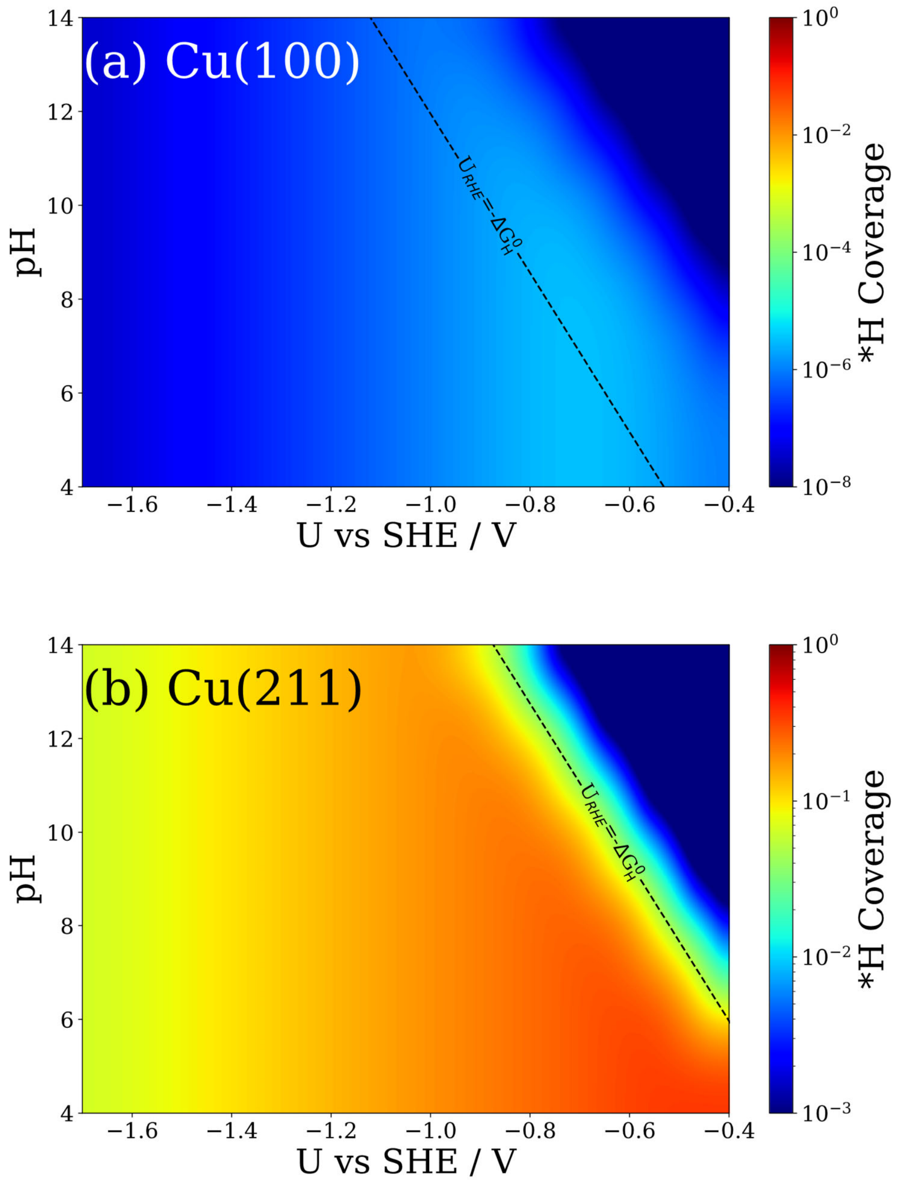

Supplementary Fig. 2. Simulated hydrogen coverage on (a) Cu(100) and (b) Cu(211) obtained using a microkinetic model. Note that the ranges covered by the colormap vary in the two panels.

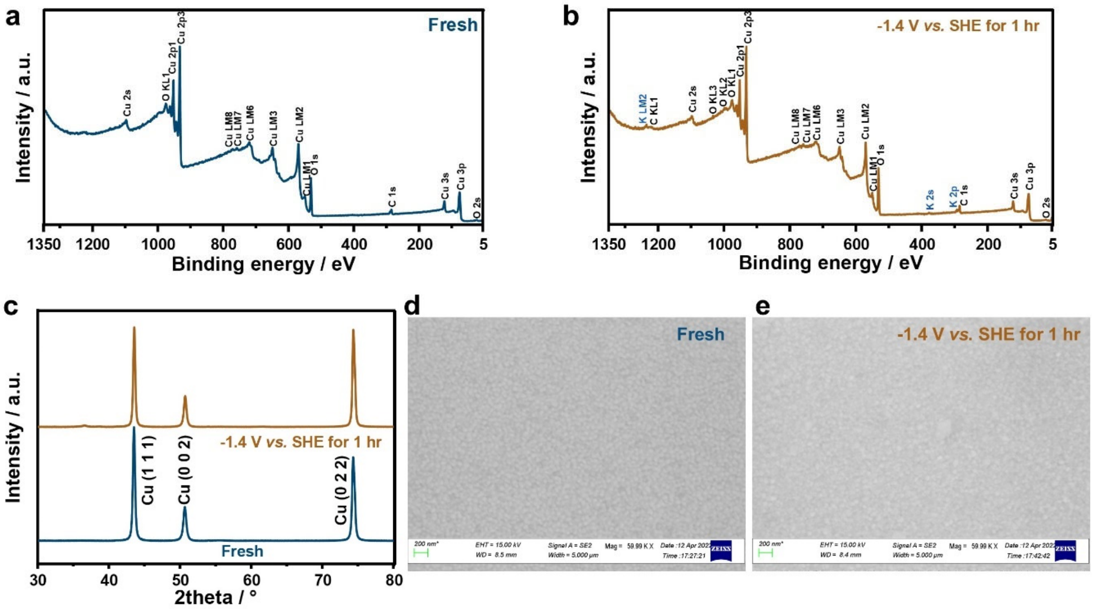

Supplementary Fig. 3. X-ray photoelectron spectroscopy (XPS) spectra in the range of 5-1350 eV for the (a) fresh Cu catalyst and (b) Cu catalyst after one hour reaction at –1.4 V *vs.* SHE in CO-saturated 0.1 M KOH electrolyte. (c) X-ray diffraction (XRD) patterns of fresh Cu catalyst and it after reaction. Scanning electron microscopy (SEM) images of the (d) fresh Cu catalyst and (e) Cu catalyst after reaction.

Boundary layer thickness Test[9]

The hydrodynamic boundary layer thickness of an electrochemical cell can be quantified by measuring the diffusion-limited current of ferricyanide reduction:

$$Fe(CN)_6^{3-} + e^- \rightarrow Fe(CN)_6^{4-}$$

Ferricyanide reduction is an ideal reaction to probe the hydrodynamic boundary layer thickness due to its electrochemical reversibility, meaning that the reduction of ferricyanide is facile such that the observed rate is limited only by mass transfer regardless of the applied overpotential. When conducting this measurement, the total ferricyanide concentration should be minimized and the supporting electrolyte should be identical to that typically employed during CO reduction. This will ensure that the fluid properties of the solution utilized to quantify the hydrodynamic boundary layer thickness accurately reflect those of the electrolytes typically employed to measure electrocatalytic activity. Furthermore, Au electrodes should be utilized to conduct the measurement to avoid Galvanic corrosion processes in which ferricyanide is the oxidizing agent. The experiment is conducted in CO saturated 0.1 M KOH with the addition of 10 mM $K_3Fe(CN)_6$. There is a potential window of ~250 mV where the observed Faradaic current can be attributed entirely to ferricyanide reduction from –0.35 V to –0.1 V *vs.* SHE. Thus, the steady state diffusion-limited current density associated with ferricyanide reduction can be measured and utilized to calculate the average hydrodynamic boundary layer thickness at the cathode surface using Fick's law:

$$\delta_{BL} = \frac{F \times D_{Fe(CN)_6^{3-}} \times C_{Fe(CN)_6^{3-}}}{j}$$

δ_{BL} is the boundary layer of the cell with an electrolyte flow rate of from 10 to 150

mL/min. F is Faraday constant and equals to 96485 C/mol; $D_{Fe(CN)_6^{3-}}$ equals to 7.26 x 10^{-6} cm^2/s;[10] $C_{Fe(CN)_6^{3-}}$ is the concentration of $Fe(CN)_6^{3-}$ and equals to 10 mM; j is Faradaic current density of Ferricyanide reduction reaction.

As shown in **Supplementary Fig. 4**, the boundary layer at 100 mL/min of our setup are 12 μm. This boundary layer thickness will be used in latter pH calculations.

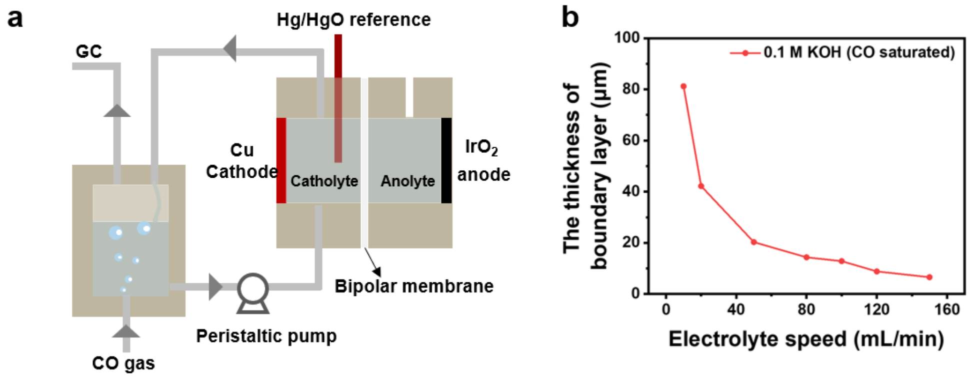

Supplementary Fig. 4. (a) Schematic illustration of custom electrochemical cell. (b) Dependence of the hydrodynamic boundary layer thicknesses at the cathode surface on the pump rate utilized to mix the catholyte determined from the measured diffusion-limited current density of ferricyanide reduction over polycrystalline Au.

We utilize BPM to reduce the diffusion of anodic ions dissolved into the solution or cathodic liquid products between the cathode and anode. Here, the cation` exchange membrane (CEM) was towards to the cathode. By using BPM, the pH of catholyte will be more stable since the consumed H^+ at the cathode can be effectively replenished by the H^+ generated through the decomposition of water on the BPM. In the design of the cell, the flow rate of the electrolyte is set at 100 mL min^{-1} to effectively reduce the thickness of the boundary layer. This also helps to prevent the pH at the interface of the BPM cathode from approaching the bulk pH. Therefore, no obvious CO_2 generation

can be observed in our experiments in $KHCO_3$ electrolyte.

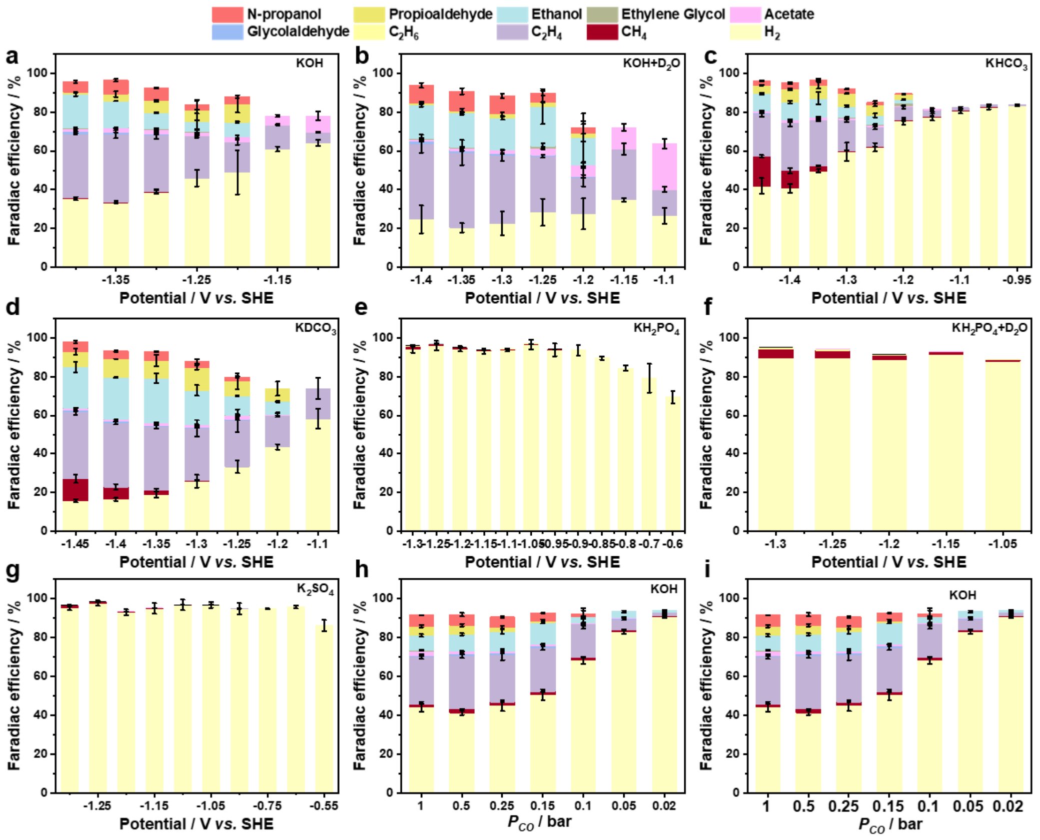

Supplementary Fig. 5. Faradaic efficiency of different products on Cu catalyst in CO-saturated 0.1 M (a) pH = 13 (KOH), (b) D_2O (KOH), (c) pH = 9 ($KHCO_3$), (d) $KDCO_3$, (e) pH = 3 (KH_2PO_4), (f) D_2O (KH_2PO_4), and (g) pH = 2 (K_2SO_4) electrolytes at different potential. Faradaic efficiency of different products on Cu catalyst in CO-saturated 0.1 M (h) KOH and (i) KH_2PO_4 at different CO partial pressure. Error bars are means ± standard deviation (n = 3 replicates).

Faradaic efficiency and partial current density calculation

The Faradaic efficiency (*FE*) of product can be calculated according to the equation:

$$FE(\%) = \frac{Q_{product}}{Q_{tot}} \times 100\% \quad \text{(S57)}$$

where $Q_{product}$ and Q_{tot} are charge transferred for product formation and charge passed through the working electrode, respectively.

Based on the equation above, the detailed calculation for *FE* of gas product (Eqn. S58) and liquid product (Eqn. S59) could be written as:

$$FE(\%) = \frac{n \times C_{g-product} \times \emptyset \times t \times \frac{P_o}{RT} \times F}{I \times t} \times 100\% \quad \text{(S58)}$$

$$FE(\%) = \frac{n \times C_{l-product} \times V \times F}{Q_{tot}} \times 100\% \quad \text{(S59)}$$

where $C_{g-product}$ and n are the concentration of gas product measured by GC and the number of electrons required for producing one molecule of the related gas product, respectively. $\emptyset$ is gas flow rate, t is the electrolysis time which can be deleted in the numerator and denominator, P_o is ambient pressure, T is absolute temperature (all experiments are performed at ambient temperature, 273.15 K), and I is current. $C_{l-product}$ is the concentration of liquid product measured by HPLC. V is the liquid value and 7 mL was used as cathodic electrolyte. Q_{tot} is the amount of charge accumulated in one hour.

The current density (*j*) of products can be calculated according to the below equation:

$$j_{product} = \frac{I \times FE_{product}}{S} \quad \text{(S60)}$$

Where *S* is the area of electrode.

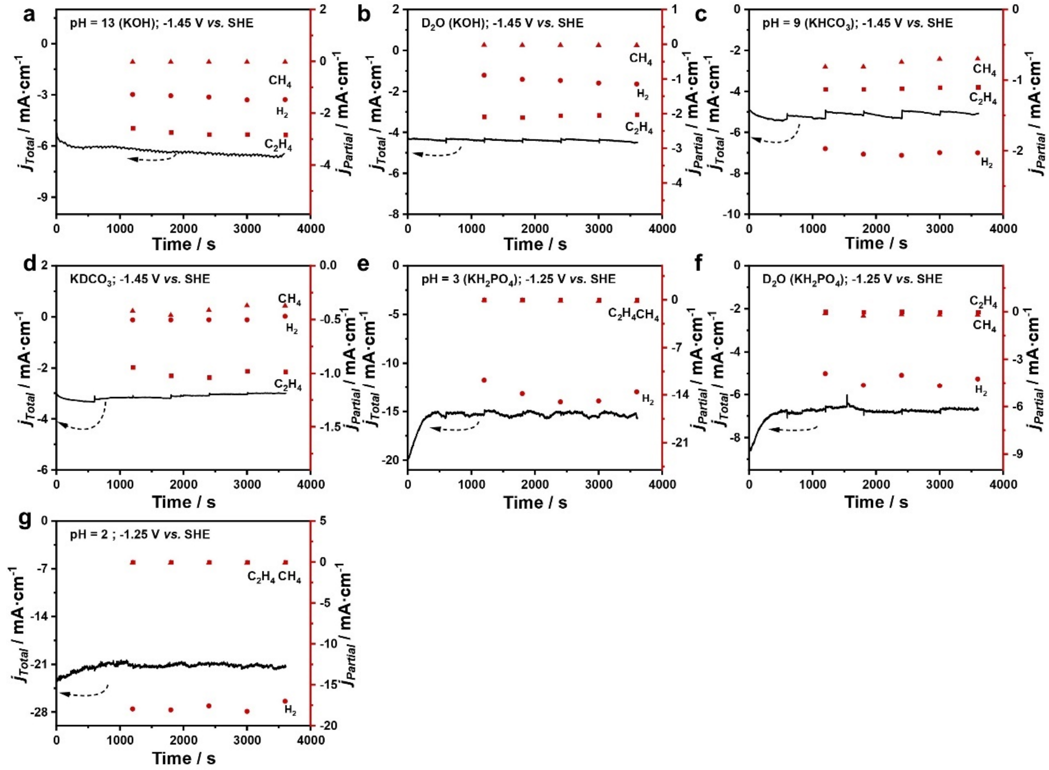

Supplementary Fig. 6. Total, H_2, CH_4 and C_2H_4 current densities versus time on Cu catalyst at CO-saturated 0.1 M (a) pH = 13 (KOH), (b) D_2O (KOH), (c) pH = 9 ($KHCO_3$), (d) $KDCO_3$, (e) pH = 3 (KH_2PO_4), (f) D_2O (KH_2PO_4), and (g) pH = 2 electrolyte during one hours, where the applying potentials are –1.45, –1.45, –1.45, –1.45, –1.25, –1.25, and –1.25 V *vs.* SHE, respectively.

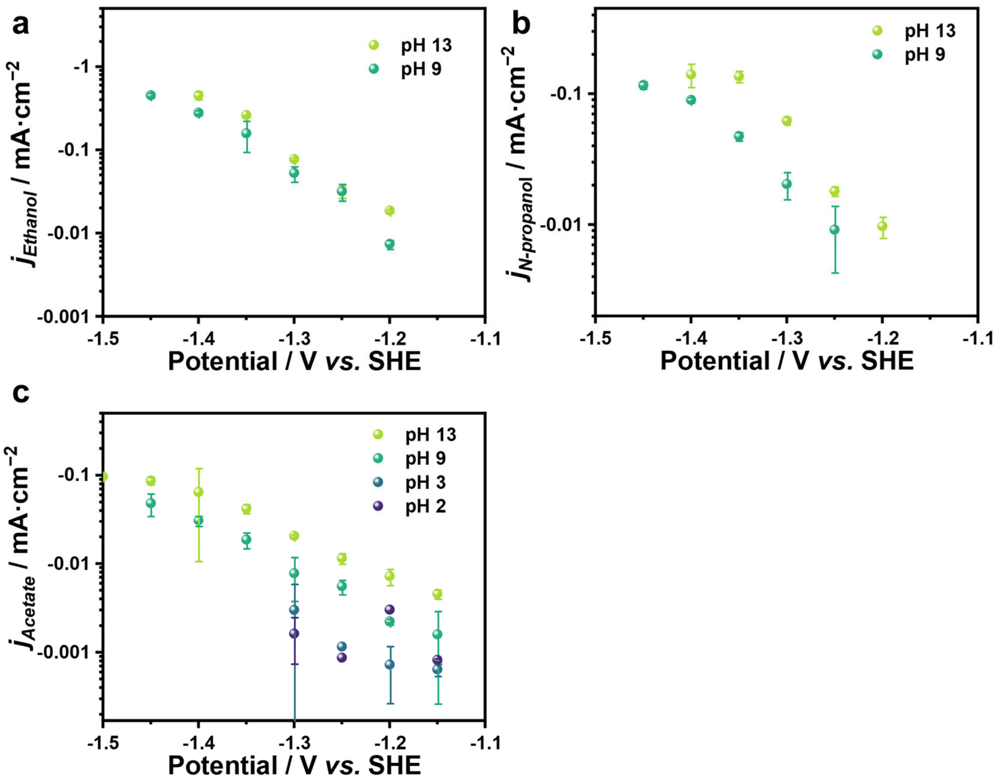

Supplementary Fig. 7. The current density of (a) ethanol, (b) n-propanol, and (c) acetate versus potential in CO-saturated electrolytes with different pH. Error bars are means ± standard deviation (n = 3 replicates).

The current density of CH_4 is even higher in an electrolyte with pH 3 than in that with pH 2 at the potential from −1.1 to −1.2 V *vs.* SHE. It may be because the presence of anionic species ($H_2PO_4^-$) from the pH 3 electrolyte might adsorb on the catalyst surface,[11,12] partially affecting the Cu electronic state. As a result, the CH_4 activity in the pH 3 solution is unexpectedly higher compared to that in the pH 2 solution. Future research on the RDS of CH_4 will require more evidence to explain the phenomena.

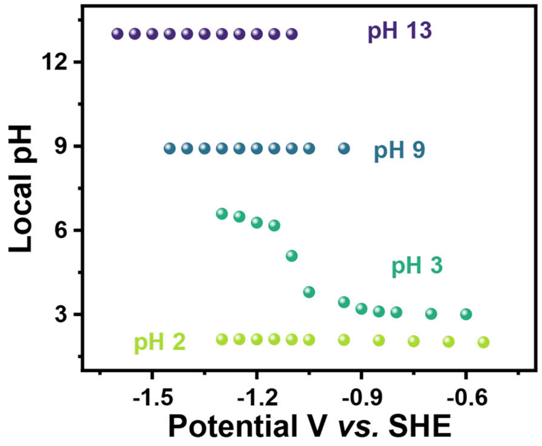

Supplementary Fig. 8. Calculated local pH in different electrolytes during COER on Cu catalyst.

Supplementary Note 3: Local pH calculation

Mass transport model

The transport of electrolyte is simulated via a 1-D model employing Poisson-Nernst-Planck equation with a boundary thickness of 12 μm.

$$\frac{dc}{dt} = -\frac{d}{dx}\left[\mathrm{D}\frac{dc}{dx} - D\frac{z_i}{k_B T} c\left(\frac{d\varphi}{dx}\right)\right] \tag{S61}$$

$$\frac{d}{dt}\left(\varepsilon \frac{d\varphi}{dx}\right) = -\sum_i z_i c_i \tag{S62}$$

where c is the concentration, D is diffusion coefficient, z_i is the charge of specie i, φ is the potential, x the distance, t the time, k_B the Boltzmann constant and T is the temperature. In Eqn. S62, ε is the permittivity of solvent. To solve these equations, we set up the Neumann boundary conditions at electrode side and Diriclet boundary conditions at solution side:

$x = 0$ (electrode surface):

$$f(CO) = \sum_i \frac{j_i^{CO}}{n_i F} \tag{S63}$$

$$f(OH^-) = \sum_i \frac{j_i^{CO}}{F} + \frac{j_{HER}}{F} \tag{S64}$$

$$\varphi^s = V + pzc \tag{S65}$$

Here, f is species flux, which could be related to current density at electrode surface via divided by the number of transfer-electrons (n_i), and Faraday constant F. All currents are from experimental data. V is the applied potential at cathode, while pzc is the potential of zero charge of Cu (–0.15 V)[13].

At x = 12 μm (solution side), we set boundary conditions as:

$$c_i = c_i^{bulk} \tag{S66}$$

$$\varphi = 0 \tag{S67}$$

In the case of acidic electrolyte, we assume that proton has priority over water to act as hydrogen source for both HER and COER, after running out of local proton, water alternatively as source of hydrogen.

During simulation, we included homogeneous reactions as below, with forward and backward rate constant from.[14,15]

$$CO_2 + OH^- \underset{k^{1+}}{\overset{k^{1-}}{\rightleftharpoons}} HCO_3^- \tag{S68}$$

$$HCO_3^- + OH^- \underset{k^{2+}}{\overset{k^{2-}}{\rightleftharpoons}} CO_3^{2-} \tag{S69}$$

$$H_2PO_4^- \underset{k^{3+}}{\overset{k^{3-}}{\rightleftharpoons}} HPO_4^{2-} + H^+ \tag{S70}$$

$$HPO_4^{2-} \underset{k^{4+}}{\overset{k^{4-}}{\rightleftharpoons}} PO_4^{3-} + H^+ \tag{S71}$$

$$OH^- + H^+ \underset{k^{5+}}{\overset{k^{5-}}{\rightleftharpoons}} H_2O \tag{S72}$$

All parameters and model set up could be found in the public repository, https://github.com/onealshu/COR_acidic.

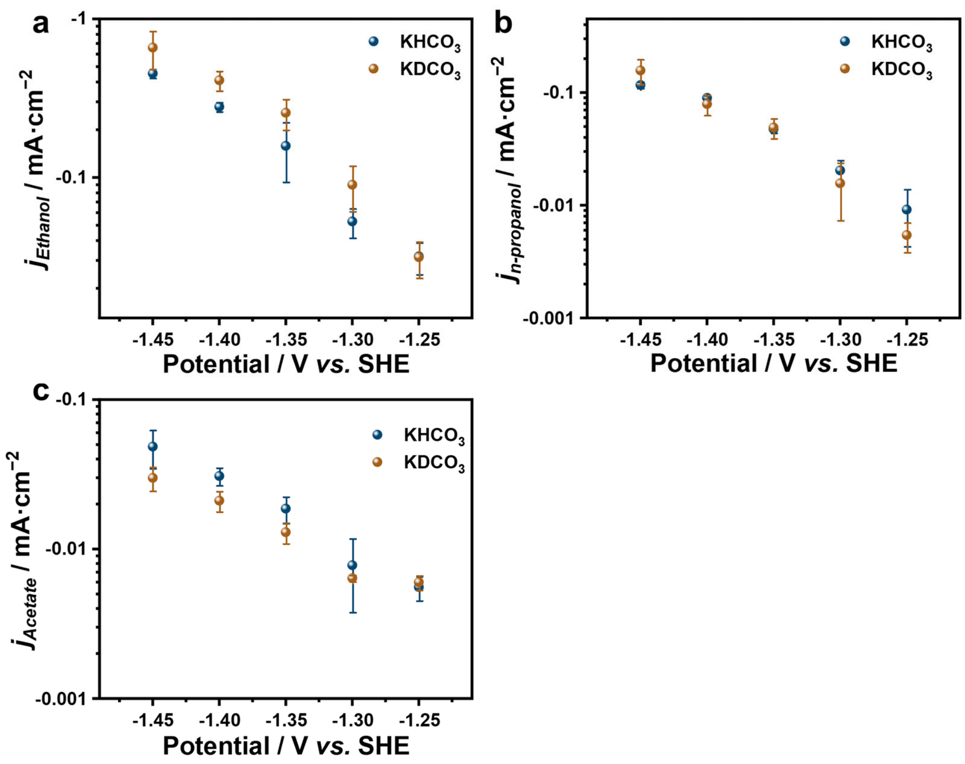

Supplementary Fig. 9. The current density of (a) ethanol, (b) n-propanol, and (c) acetate versus potential in CO-saturated 0.1 M $KHCO_3$ and $KDCO_3$ electrolytes. Error bars are means ± standard deviation (n = 3 replicates).

These C_{2+} products exhibits slightly different KIE changes for acetate (KIE > 1) and ethanol (KIE < 1). There may be two potential explanations here. Firstly, C_{2+} products share the same RDS, which is the *CO-*CO coupling. However, after the formation of the $*C_2O_2^{\delta-}$ intermediate, the subsequent selectivity-determining steps among different C_{2+} products are influenced by H/D exchange. For instance, when OH^- is replaced by OD^-, the crucial step for acetate formation in which ketene (an important intermediate derived from acetate) is attacked by OH^- is slowed down. This results in a KIE < 1 for acetate. At the same time, more ketene can be reduced to ethanol, leading to a KIE > 1. However, the overall KIE value for C_{2+} remains ~1 (Fig. 2a). The second potential explanation is that different carbon-containing products indeed have different RDS. Since our catalyst mainly produces C_2H_4 as the primary C_{2+} product, it becomes challenging to evaluate other carbon-containing products based on the existing data. To individually identify the RDS for each carbon-containing product, it is necessary to first

identify the catalyst that favors the production of that product. This approach would minimize the selectivity differences caused by testing conditions, ensuring accurate analysis of the kinetic data.

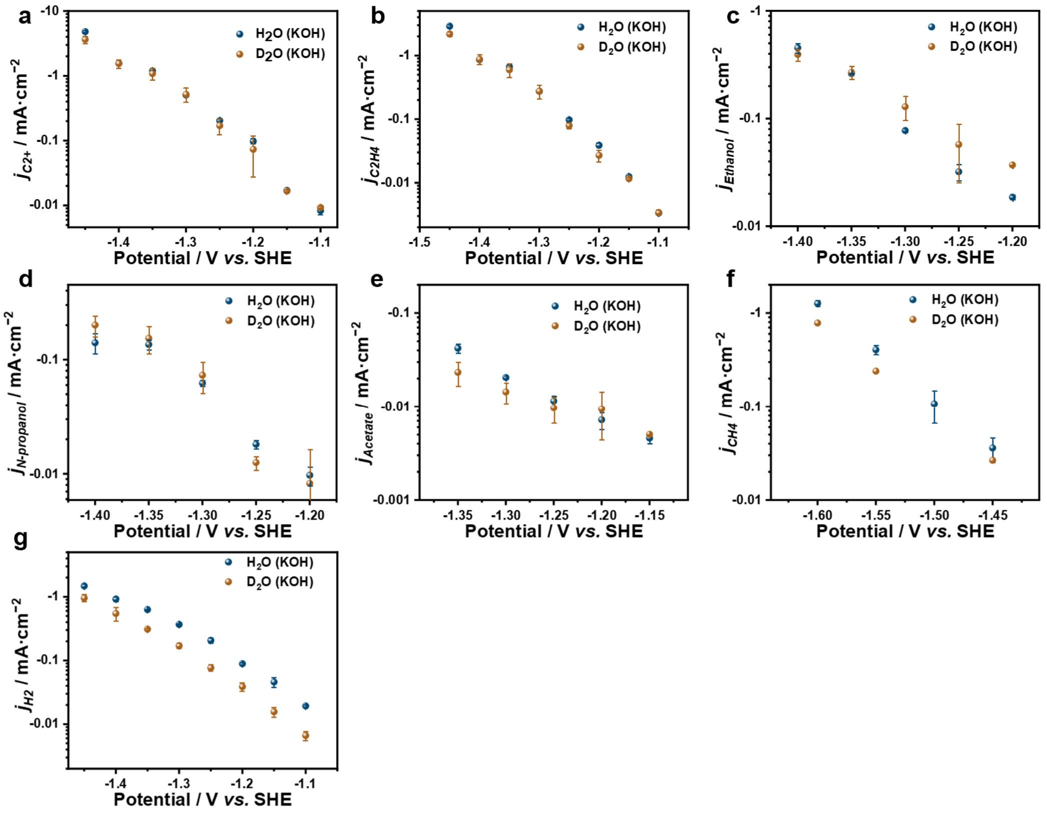

Supplementary Fig. 10. The current density of (a) C_{2+}, (b) C_2H_4, (c) ethanol, (d) n-propanol, (e) acetate, (f) CH_4, and (g) H_2 versus potential in CO-saturated 0.1 M KOH electrolytes in H_2O and D_2O. Error bars are means ± standard deviation (n = 3 replicates).

After the replacement of H with D atoms in the electrolyte, D atoms with a higher mass fraction will exhibit slower adsorption and desorption during the reaction compared to hydrogen atoms. In reactions involving proton-related RDS or preceding steps, such as the HER and COER to CH_4, there will be a noticeable decrease in activity. However, for reactions that do not involve protons in the RDS or preceding steps, the slower-reacting D atoms will not exhibit a significant impact.

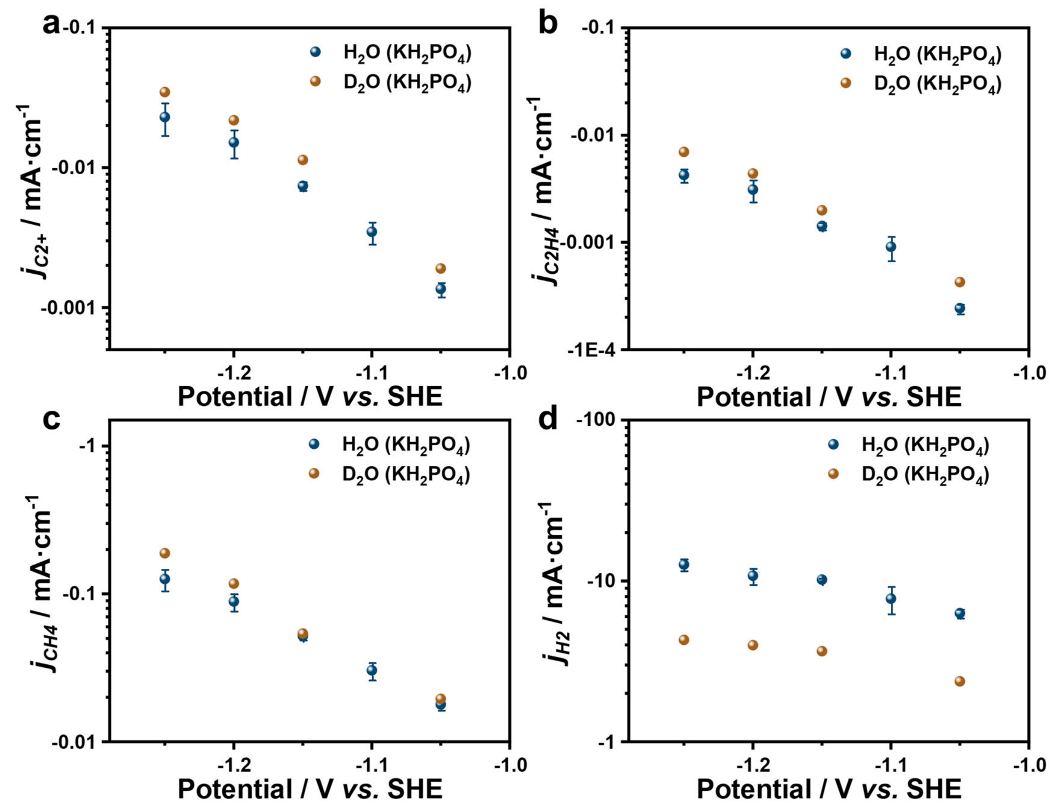

Supplementary Fig. 11. The current density of (a) C_{2+}, (b) C_2H_4, (c) CH_4, and (d) H_2 versus potential in CO-saturated 0.1 M KH_2PO_4 electrolytes in H_2O and D_2O. Error bars are means ± standard deviation (n = 3 replicates).

In the solution at pH 3, the current density of CH_4 remained constant with the substitution of deuterium atoms at potential from −1.05 to −1.15 V *vs.* SHE, while it increased instead at −1.20 to −1.25 V *vs.* SHE. This is inconsistent with the decrease trends observed in solutions of pH 13 and 9 (Fig 2c and Supplementary Fig. 10f). As discussed in the main text, $H_2PO_4^-$ anion will adsorb over Cu surface at reaction condition, might result in a KIE ⩽ 1, even though its KIE should be greater than 1.[16,17] Considering that there is lack of sufficient evidence to explain the anomalous behavior, further study should be required according to the CH4 mechanism study. Because it not being the focus of this work, further discussion on this topic is omitted.

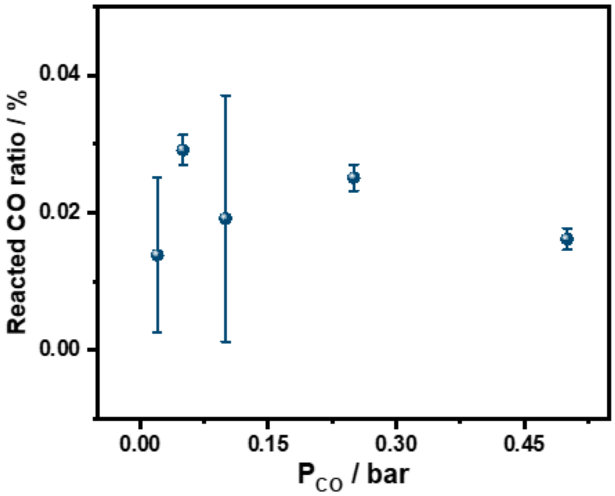

Supplementary Fig. 12. The conversion rate of CO under different pressures. All those data are collected in CO-saturated 0.1 M KOH electrolyte at –1.3 V *vs.* SHE. Error bars are means ± standard deviation (n = 3 replicates).

Supplementary Note 4: The non-linear fit based on the rate expression.

Supplementary Table 1. The non-linear fit formulars based on the rate expression and the related fitting parameters in Fig. 3.

Step	Non-linear fit formular[a]	n_{CO}	a_i	b_i	R^2
A2	$j_{C2+} = \frac{a_i P_{CO}^2}{(1+b_i P_{CO})^2}$	2 ~ 0	132.9	17.3	0.9884
B2	$j_{C2+} = \frac{a_i P_{CO}^2}{1+b_i P_{CO}}$	2 ~ 1	29796620.8	5772584<br>4.3	0.0856
C2 and D2	$j_{C2+} = \frac{a_i P_{CO}}{1+b_i P_{CO}}$	1 ~ 0	2.9	6.1	0.9692
E3 and F3	$j_{C2+} = \frac{a_i P_{CO}}{(b_i P_{CO}+1)^2}$	1 ~ −1	2.2	1.4	0.9843

[a]a_i and b_i represents fitting parameters, which are constants and their specific physical means can be found in Supplementary Note 2. According to Supplementary Note 2, we can obtain the rate expression of each RDS. Simplifying those rate expressions, seven types of formulars are observed and utilized for non-linear fitting. The fitting parameters are shown in Supplementary Table 1.

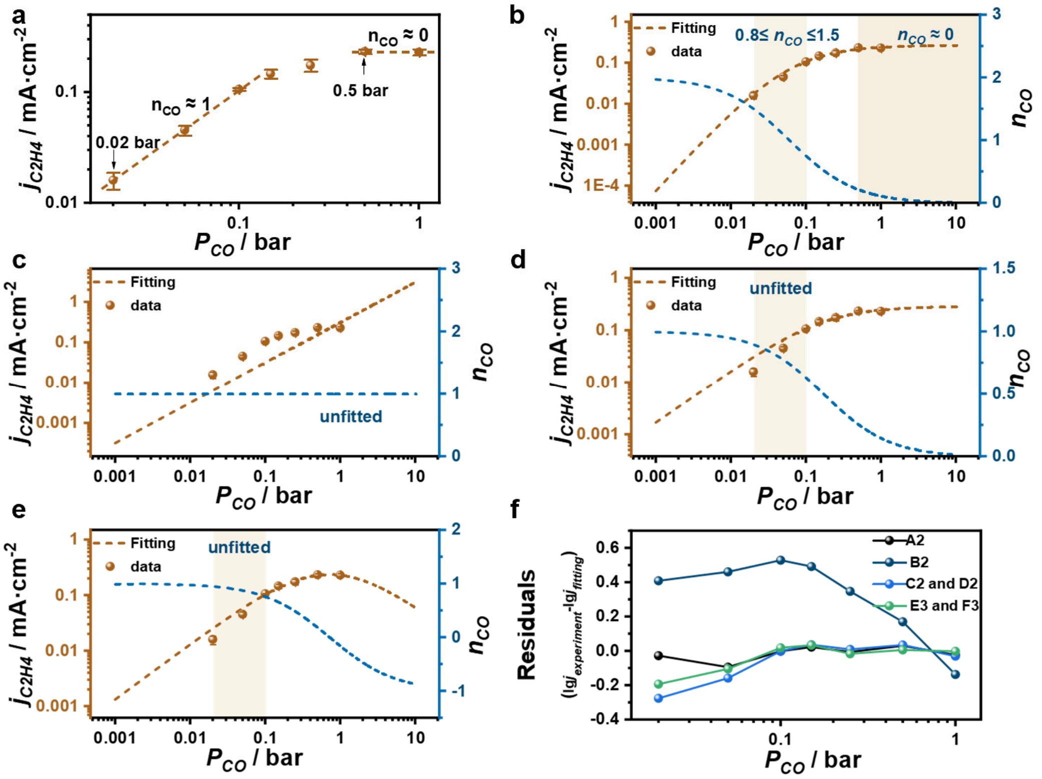

Supplementary Fig. 13. The current density of C_2H_4 versus CO partial pressure for (a) interval linear and (b-e) nonlinear data fitting as well as their residuals (f). The equations used for fitting (b-e) are the theoretical rate expressions corresponding to different mechanisms (see Supplementary Table 2 for all the fitting parameters). All those data are collected in CO-saturated 0.1 M KOH electrolyte at –1.3 V *vs.* SHE. Error bars are means ± standard deviation (n = 3 replicates).

Supplementary Table 2. The non-linear fit formulars based on the rate expression and the related fitting parameters in **Supplementary Fig. 13**.

Step	Non-linear fit formular[a]	n_{CO}	a_i	b_i	R^2
A2	$j_{C2+}=\frac{a_i P_{CO}^2}{(1+b_i P_{CO})^2}$	2 ~ 0	75.9	16.7	0.9864
B2	$j_{C2+}=\frac{a_i P_{CO}^2}{1+b_i P_{CO}}$	2 ~ 1	1742.0	5522.5	0.1082
C2 and D2	$j_{C2+}=\frac{a_i P_{CO}}{1+b_i P_{CO}}$	1 ~ 0	1.7	5.9	0.9690
E3 and F3	$j_{C2+}=\frac{a_i P_{CO}}{(b_i P_{CO}+1)^2}$	1 ~ −1	2.2	1.4	0.9900

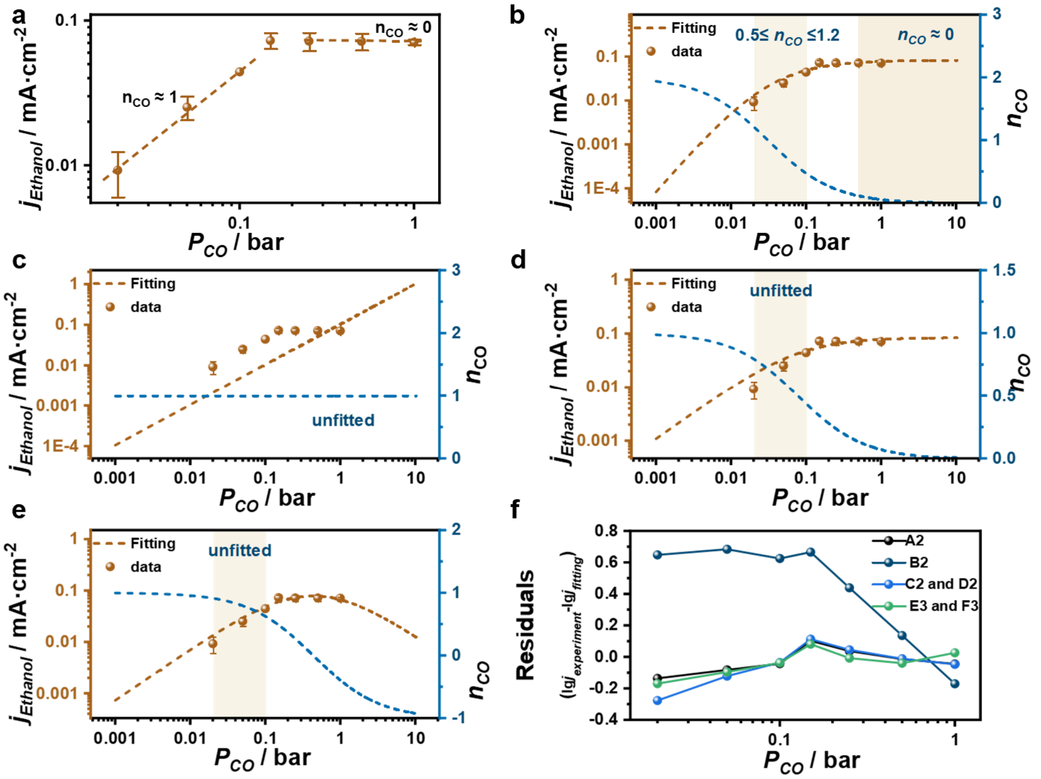

Supplementary Fig. 14. The current density of ethanol versus CO partial pressure for (a) interval linear and (b-e) nonlinear data fitting as well as their residuals (f). The equations used for fitting (b-e) are the theoretical rate expressions corresponding to different mechanisms (see Supplementary Table 3 for all the fitting parameters). All those data are collected in CO-saturated 0.1 M KOH electrolyte at –1.3 V *vs.* SHE. Error bars are means ± standard deviation (n = 3 replicates).

Supplementary Table 3. The non-linear fit formulars based on the rate expression and the related fitting parameters in **Supplementary Fig. 14**.

Step	Non-linear fit formular[a]	n_{CO}	a_i	b_i	R^2
A2	$j_{C2+} = \frac{a_i P_{CO}^2}{(1+b_i P_{CO})^2}$	2 ~ 0	87.9	32.4	0.9011
B2	$j_{C2+} = \frac{a_i P_{CO}^2}{1+b_i P_{CO}}$	2 ~ 1	453.7	4316.5	-1.0240
C2 and D2	$j_{C2+} = \frac{a_i P_{CO}}{1+b_i P_{CO}}$	1 ~ 0	1.1	12.9	0.8697
E3 and F3	$j_{C2+} = \frac{a_i P_{CO}}{(b_i P_{CO}+1)^2}$	1 ~ −1	0.7	2.3	0.9357

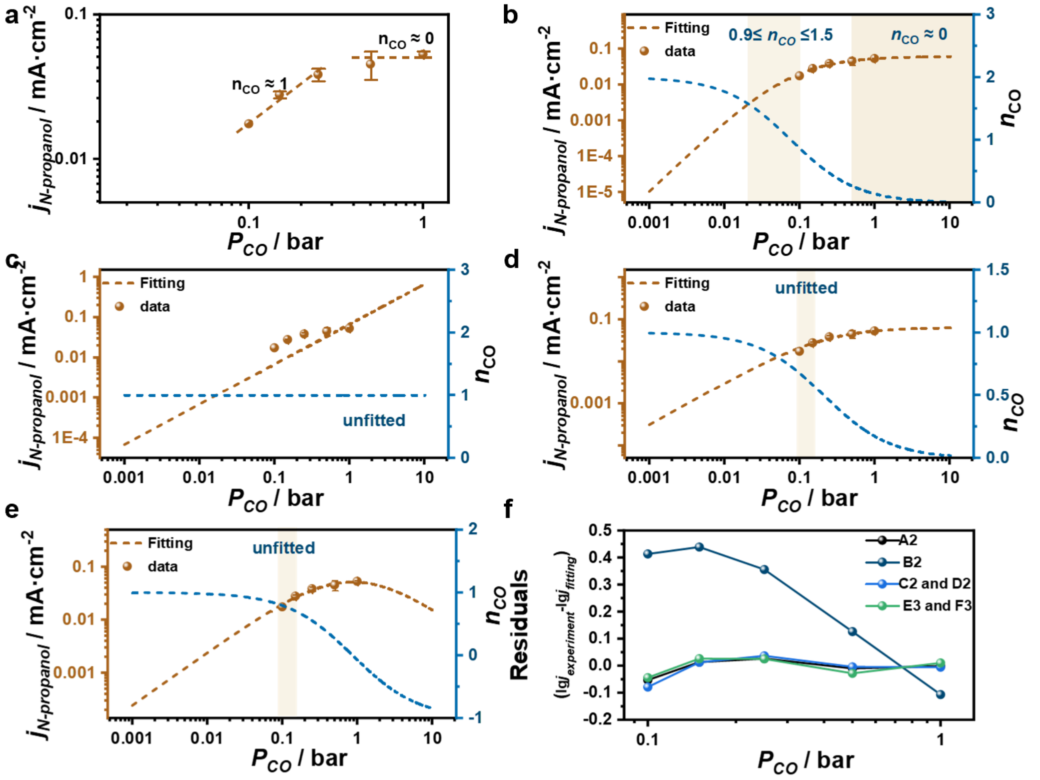

Supplementary Fig. 15. The current density of n-propanol versus CO partial pressure for (a) interval linear and (b-e) nonlinear data fitting as well as their residuals (f). The equations used for fitting (b-e) are the theoretical rate expressions corresponding to different mechanisms (see Supplementary Table 4 for all the fitting parameters). All those data are collected in CO-saturated 0.1 M KOH electrolyte at –1.3 V *vs.* SHE. Error bars are means ± standard deviation (n = 3 replicates).

Supplementary Table 4. The non-linear fit formulars based on the rate expression and the related fitting parameters in **Supplementary Fig. 15**.

Step	Non-linear fit formular[a]	n_{CO}	a_i	b_i	R^2
A2	$j_{C2+}=\frac{a_i P_{CO}^2}{(1+b_i P_{CO})^2}$	2 ~ 0	10.5	13.0	0.9844
B2	$j_{C2+}=\frac{a_i P_{CO}^2}{1+b_i P_{CO}}$	2 ~ 1	146.8	2163.1	-0.5689
C2 and D2	$j_{C2+}=\frac{a_i P_{CO}}{1+b_i P_{CO}}$	1 ~ 0	0.3	4.7	0.9710
E3 and F3	$j_{C2+}=\frac{a_i P_{CO}}{(b_i P_{CO}+1)^2}$	1 ~ −1	0.2	1.2	0.9726

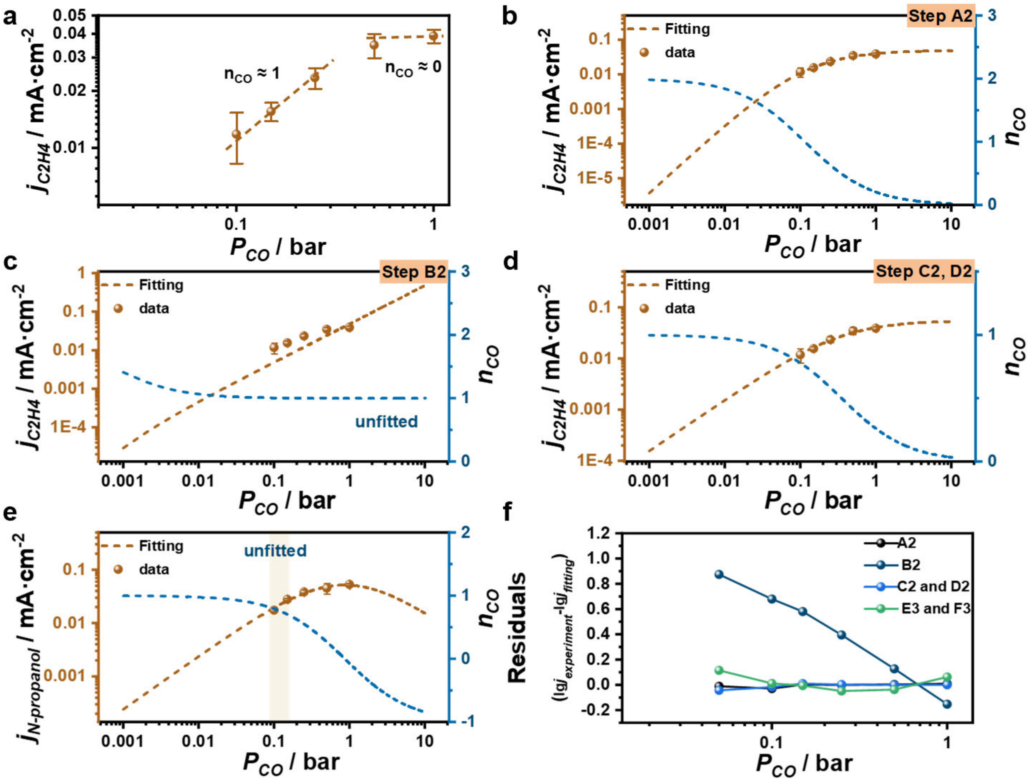

Supplementary Fig. 16. The current density of C_2H_4 versus CO partial pressure for (a) interval linear and (b-e) nonlinear data fitting as well as their residuals (f). The equations used for fitting (b-e) are the theoretical rate expressions corresponding to different mechanisms (see Supplementary Table 5 for all the fitting parameters). All those data are collected in CO-saturated 0.1 M KOH electrolyte at –1.1 V *vs.* SHE. Error bars are means ± standard deviation (n = 3 replicates).

Supplementary Table 5. The non-linear fit formulars based on the rate expression and the related fitting parameters in **Supplementary Fig. 16**.

Step	Non-linear fit formular[a]	n_{CO}	a_i	b_i	R^2
A2	$j_{C2+}=\frac{a_iP_{CO}^2}{(1+b_iP_{CO})^2}$	2 ~ 0	3.7	8.7	0.9905
B2	$j_{C2+}=\frac{a_iP_{CO}^2}{1+b_iP_{CO}}$	2 ~ 1	70.7	1448.6	0.2840
C2 and D2	$j_{C2+}=\frac{a_iP_{CO}}{1+b_iP_{CO}}$	1 ~ 0	0.2	2.8	0.9750
E3 and F3	$j_{C2+}=\frac{a_iP_{CO}}{(b_iP_{CO}+1)^2}$	1 ~ −1	0.1	0.9	0.9918

To further exploring the effect of potential, we also conducted experiments at –1.1 V vs. SHE. As it is challenging to detect liquid products at this potential, the main C_{2+} products are ethylene, as shown in Supplementary Fig. 16. Here, the CO partial pressure data can be fitted for most mechanisms, except for Step B2, because it is hard to go lower CO pressure with detectable C_2H_4 concentration.

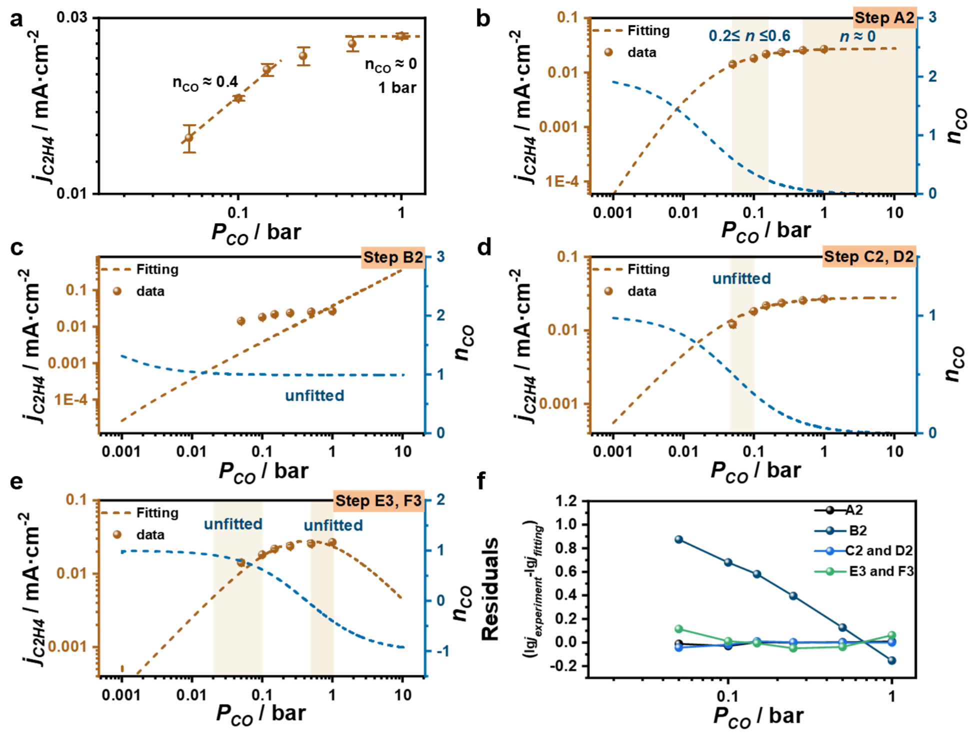

Supplementary Fig. 17. The current density of C_2H_4 versus CO partial pressure for (a) interval linear and (b-e) nonlinear data fitting as well as their residuals (f). The equations used for fitting (b-e) are the theoretical rate expressions corresponding to different mechanisms (see Supplementary Table 6 for all the fitting parameters). All those data are collected in CO-saturated 0.1 M KH_2PO_4 electrolyte (pH 3) at –1.3 V *vs.* SHE. Error bars are means ± standard deviation (n = 3 replicates).

Supplementary Table 6. The non-linear fit formulars based on the rate expression and the related fitting parameters in **Supplementary Fig. 17**.

Step	Non-linear fit formular[a]	n_{CO}	a_i	b_i	R^2
A2	$j_{C2+} = \frac{a_i P_{CO}^2}{(1+b_i P_{CO})^2}$	2 ~ 0	80.0	54.1	0.9857
B2	$j_{C2+} = \frac{a_i P_{CO}^2}{1+b_i P_{CO}}$	2 ~ 1	110.1	2882.7	-3.0470
C2 and D2	$j_{C2+} = \frac{a_i P_{CO}}{1+b_i P_{CO}}$	1 ~ 0	0.6	20.7	0.9959
E3 and F3	$j_{C2+} = \frac{a_i P_{CO}}{(b_i P_{CO}+1)^2}$	1 ~ −1	0.3	2.4	0.8029

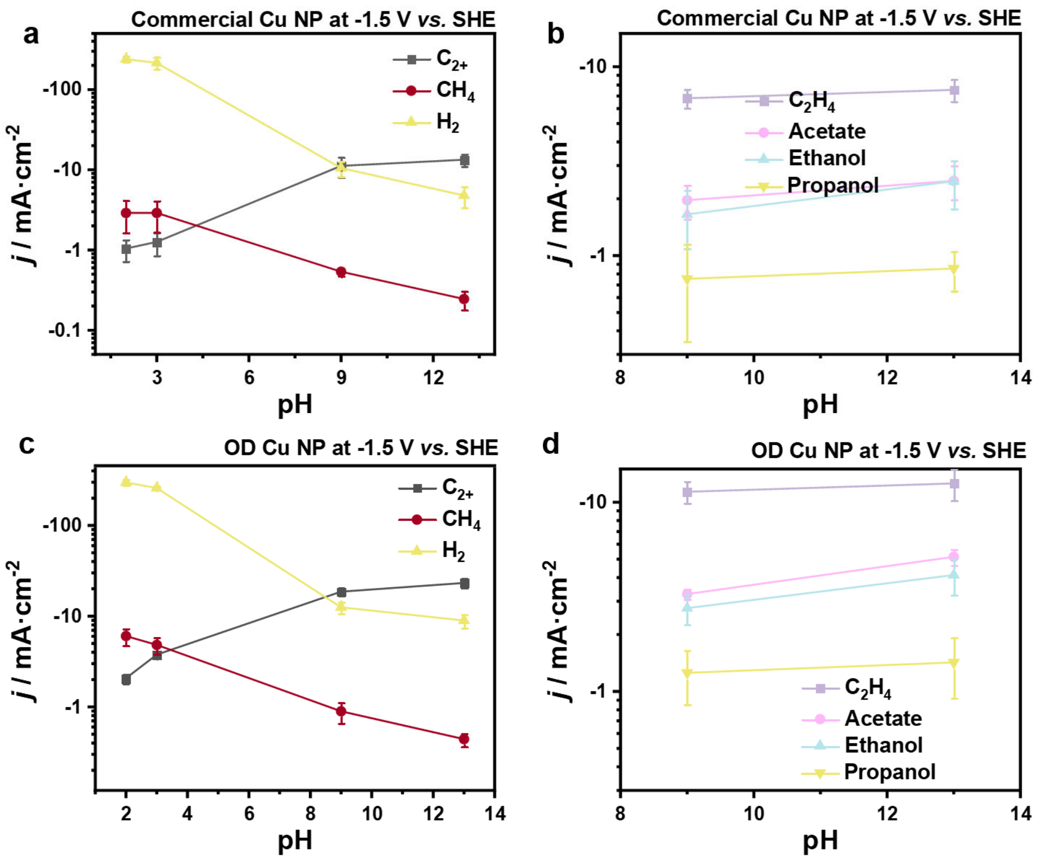

Supplementary Fig. 18. The current density of different products at different pH for (a and b) commercial Cu nanoparticles (Cu NP 40~60 nm, Sigma-Aldrich) and (c and d) oxide-derived Cu (OD-Cu) at –1.5 V *vs.* SHE in flow cell. Considering that a higher potential is required for CH_4 generation, and that the same voltage was employed in our previous CO reaction order experiments in H-cell, –1.5 V vs. SHE was applied here. The application of a flow cell in those experiments is primarily motivated by two considerations. Firstly, it is difficult to measure this type of powdered catalyst in our H-cell, as it is challenging to prepare such catalyst films. Additionally, it is interesting to investigate whether the mechanism would be different under higher current density or different cell configuration. For specific flow cell details, please refer to our previous the article.[18] OD-Cu is prepared by subjecting commercial Cu nanoparticles to a reduction process under in-situ negative potential after being treated in an O_2 atmosphere at 300 °C for 1 hour. Error bars are means ± standard deviation (n = 3 replicates).

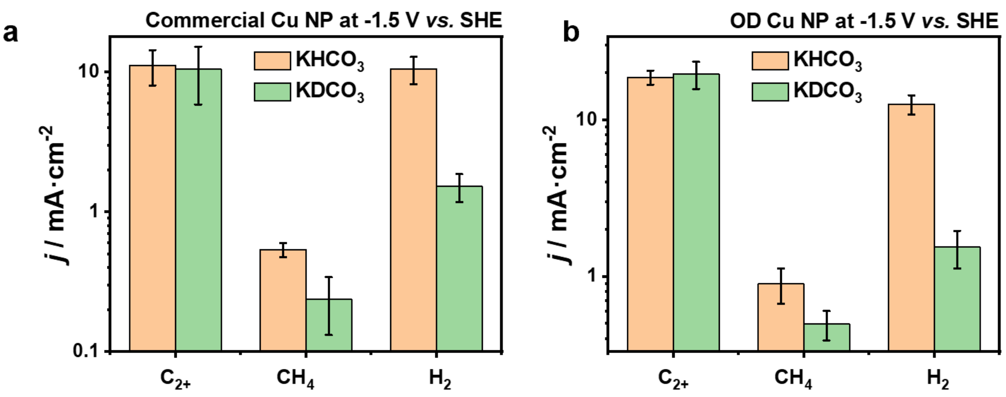

Supplementary Fig. 19. The current density of C_{2+} products on (a) commercial Cu and (b) OD Cu NP in CO-saturated 0.1 M $KHCO_3$ and $KDCO_3$ electrolytes at –1.5 V *vs.* SHE in flow cell. Error bars are means ± standard deviation (n = 3 replicates).

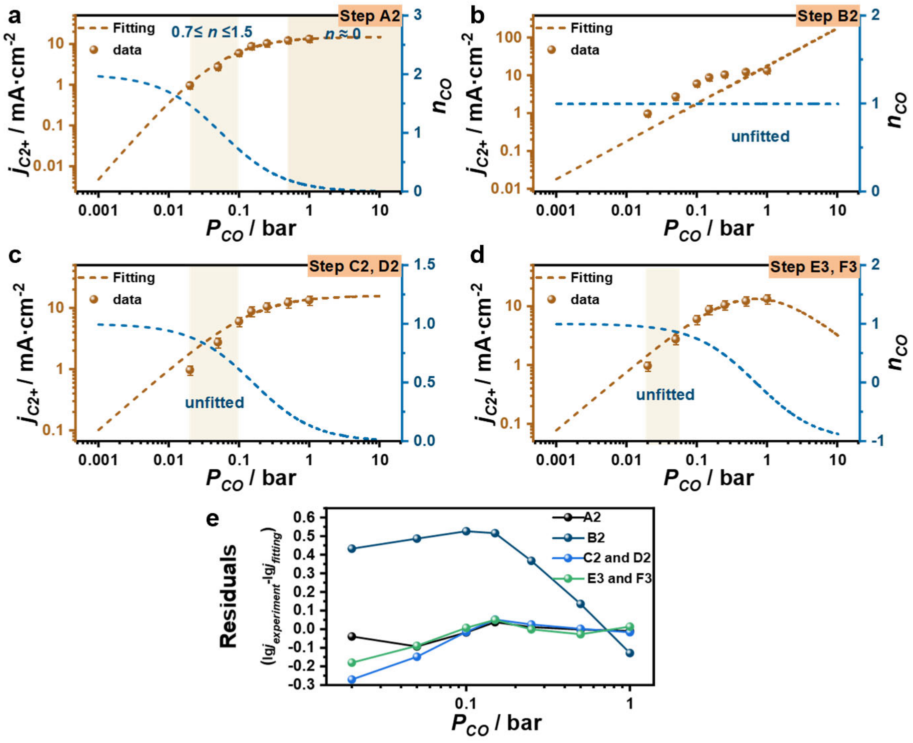

Supplementary Fig. 20. The current density of C_{2+} products versus CO partial pressure for Cu NP with nonlinear data fitting (a-d) as well as their residuals (e). The equations used for fitting are the theoretical rate expressions corresponding to different mechanisms (see Supplementary Table 7 for all the fitting parameters). All those data are collected in CO-saturated 0.1 M KOH electrolyte at –1.5 V *vs.* SHE in flow cell. Error bars are means ± standard deviation (n = 3 replicates).

Supplementary Table 7. The non-linear fit formulars based on the rate expression and the related fitting parameters in **Supplementary Fig. 20**.

Step	Non-linear fit formular[a]	n_{CO}	a_i	b_i	R^2
A2	$j_{C2+}=\frac{a_i P_{CO}^2}{(1+b_i P_{CO})^2}$	2 ~ 0	4913.7	17.9	0.9910
B2	$j_{C2+}=\frac{a_i P_{CO}^2}{1+b_i P_{CO}}$	2 ~ 1	29048429<br>35.4	16181249<br>1.2	0.0550
C2 and D2	$j_{C2+}=\frac{a_i P_{CO}}{1+b_i P_{CO}}$	1 ~ 0	102.0	6.3	0.9731
E3 and F3	$j_{C2+}=\frac{a_i P_{CO}}{(b_i P_{CO}+1)^2}$	1 ~ −1	77.8	1.5	0.9823

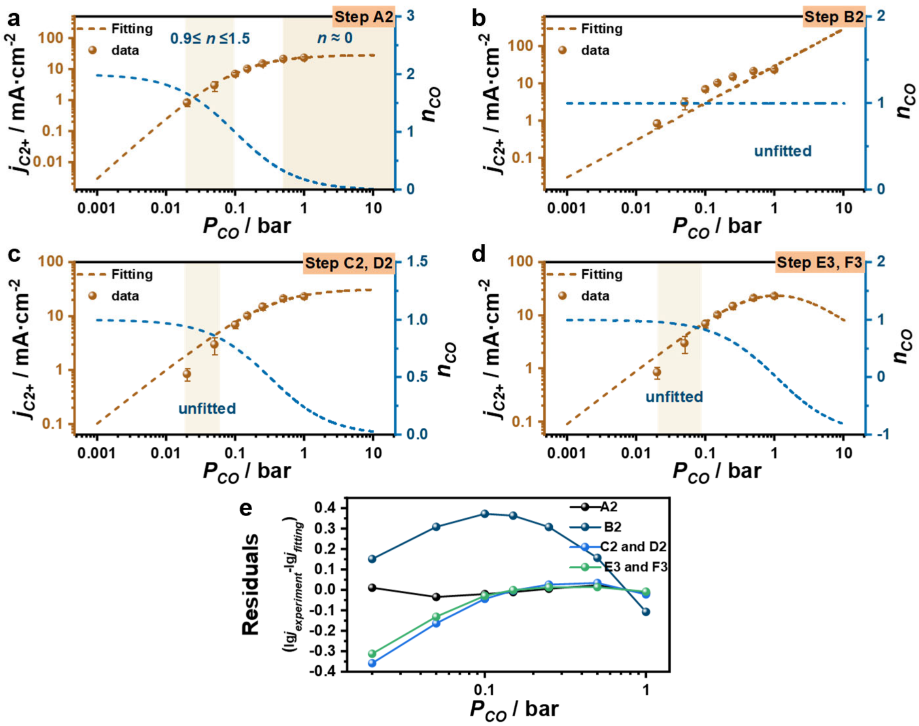

Supplementary Fig. 21. The current density of C_{2+} products versus CO partial pressure for OD-Cu with nonlinear data fitting (a-d) as well as their residuals (e). The equations used for fitting are the theoretical rate expressions corresponding to different mechanisms (see Supplementary Table 8 for all the fitting parameters). All those data are collected in CO-saturated 0.1 M KOH electrolyte at –1.5 V *vs.* SHE in flow cell. Error bars are means ± standard deviation (n = 3 replicates).

Supplementary Table 8. The non-linear fit formulars based on the rate expression and the related fitting parameters in **Supplementary Fig. 21**.

Step	Non-linear fit formular[a]	n_{CO}	a_i	b_i	R^2
A2	$j_{C2+} = \frac{a_i P_{CO}^2}{(1+b_i P_{CO})^2}$	2 ~ 0	2976.4	10.1	0.9953
B2	$j_{C2+} = \frac{a_i P_{CO}^2}{1+b_i P_{CO}}$	2 ~ 1	59614440<br>90.6	20007386<br>2.7	0.5689
C2 and D2	$j_{C2+} = \frac{a_i P_{CO}}{1+b_i P_{CO}}$	1 ~ 0	102.4	3.2	0.9813
E3 and F3	$j_{C2+} = \frac{a_i P_{CO}}{(b_i P_{CO}+1)^2}$	1 ~ −1	89.9	0.95	0.9934

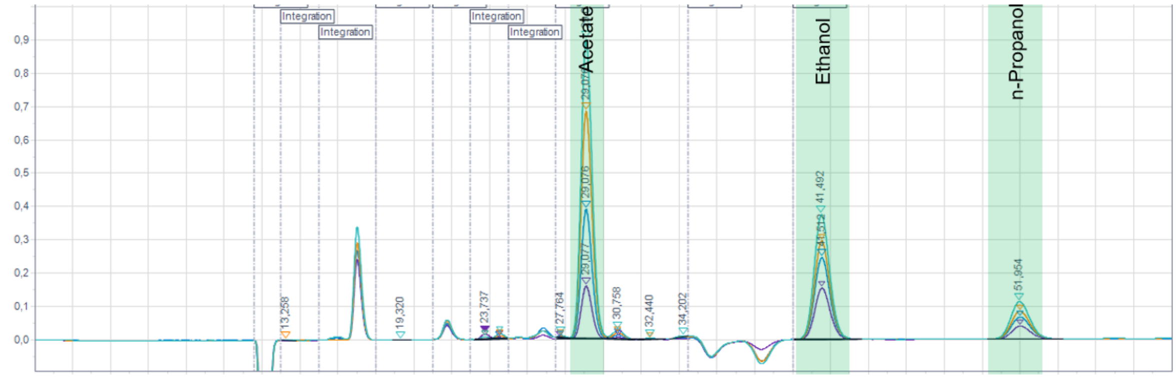

Supplementary Fig. 22. Some raw HPLC spectra of catholyte sample.

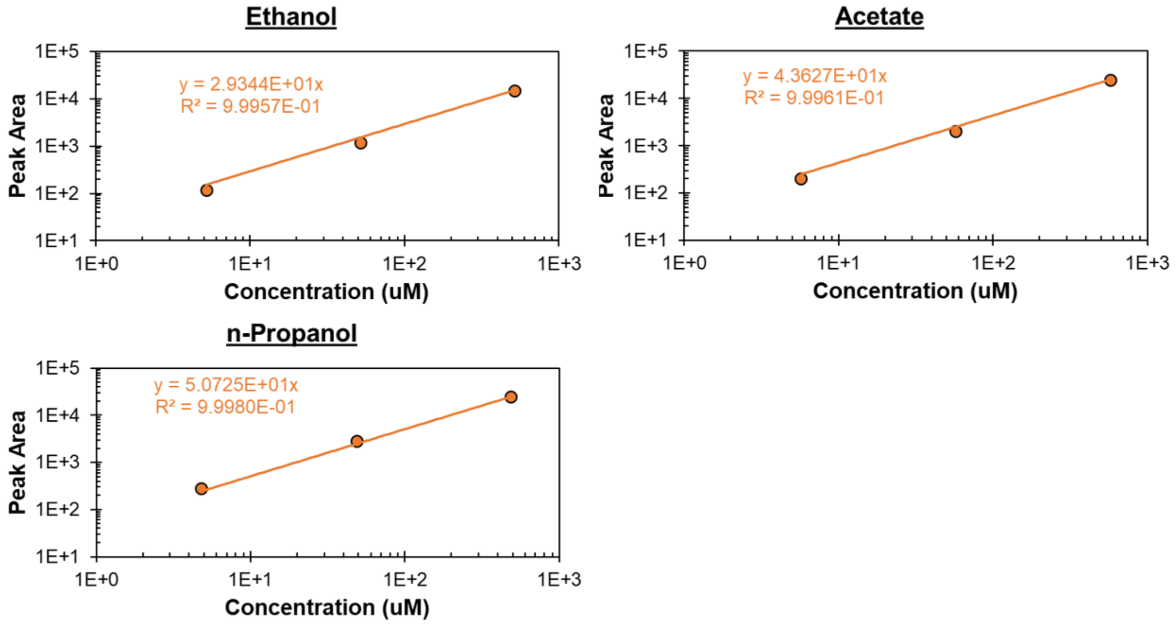

Supplementary Fig. 23. The calibration curves of liquid products.

Supplementary References

1. Kastlunger, G., Lindgren, P. & Peterson, A. A. Controlled-potential simulation of elementary electrochemical reactions: proton discharge on metal surfaces. *J. Phys. Chem. C* **122**, 12771-12781 (2018).
2. Mortensen, J. J., Hansen, L. B. & Jacobsen, K. W. Real-space grid implementation of the projector augmented wave method. *Phys. Rev. B* **71**, 035109 (2005).
3. Enkovaara, J.*, et al.* Electronic structure calculations with GPAW: A real-space implementation of the projector augmented-wave method. *J. Condens. Matter Phys.* **22**, 253202 (2010).
4. Kastlunger, G.*, et al.* Using pH dependence to understand mechanisms in electrochemical CO reduction. *ACS Catal.* **12**, 4344-4357 (2022).
5. Medford, A. J.*, et al.* CatMAP: a software package for descriptor-based microkinetic mapping of catalytic trends. *Catal. Lett.* **145**, 794-807 (2015).
6. Sandberg, R. B., Montoya, J. H., Chan, K. & Nørskov, J. K. CO-CO coupling on Cu facets: Coverage, strain and field effects. *Surf. Sci.* **654**, 56-62 (2016).
7. Liu, X., Xiao, J., Peng, H., Hong, X., Chan, K. & Nørskov, J. K. Understanding trends in electrochemical carbon dioxide reduction rates. *Nat. Commun.* **8**, 15438 (2017).
8. Trasatti, S. The absolute electrode potential: an explanatory note (Recommendations 1986). *Pure Appl. Chem.* **58**, 955-966 (1986).
9. Clark, E. L.*, et al.* Standards and Protocols for Data Acquisition and Reporting for Studies of the Electrochemical Reduction of Carbon Dioxide. *ACS Catal.* **8**, 6560-6570 (2018).
10. Konopka, S. J. & McDuffie, B. Diffusion coefficients of ferri- and ferrocyanide ions in aqueous media, using twin-electrode thin-layer electrochemistry. *Anal. Chem.* **42**, 1741-1746 (1970).
11. Niaura, G., Gaigalas, A. K. & Vilker, V. L. Surface-enhanced Raman spectroscopy of phosphate anions: adsorption on silver, gold, and copper electrodes. *J. Phys. Chem. B* **101**, 9250-9262 (1997).
12. Hochfilzer, D.*, et al.* Transients in electrochemical CO reduction explained by mass transport of buffers. *ACS Catal.* **12**, 5155-5161 (2022).
13. Soliman, K. Electrochemical behaviour of pseudomorphic Ag and Cu monolayers on well-ordered single crystal surfaces and of Ir (210) nanofacets.). Universität Ulm (2008).
14. Schulz, K. G., Riebesell, U., Rost, B., Thoms, S. & Zeebe, R. E. Determination of the rate constants for the carbon dioxide to bicarbonate inter-conversion in pH-buffered seawater systems. *Mar. Chem.* **100**, 53-65 (2006).
15. Hashiba, H.*, et al.* Effects of electrolyte buffer capacity on surface reactant species and the reaction rate of CO_2 in electrochemical CO_2 reduction. *J. Phys. Chem. C* **122**, 3719-3726 (2018).
16. Schreier, M., Yoon, Y., Jackson, M. N. & Surendranath, Y. Competition between H and CO for active sites governs copper-mediated electrosynthesis of hydrocarbon fuels. *Angew. Chem. Int. Ed.* **57**, 10221-10225 (2018).
17. Li, J.*, et al.* Electrokinetic and in situ spectroscopic investigations of CO electrochemical reduction on copper. *Nat. Commun.* **12**, 3264 (2021).
18. Hendrik, H. H.*, et al.* Mechanism for acetate formation in electrochemical CO_2 reduction on Cu: Selectivity with potential, pH, and nanostructuring. *Energy Environ. Sci.* **15**, 3978-3990 (2022).
